# Physiologic Course of Female Reproductive Function: A Molecular Look into the Prologue of Life

**DOI:** 10.1155/2015/715735

**Published:** 2015-12-01

**Authors:** Joselyn Rojas, Mervin Chávez-Castillo, Luis Carlos Olivar, María Calvo, José Mejías, Milagros Rojas, Jessenia Morillo, Valmore Bermúdez

**Affiliations:** Endocrine-Metabolic Research Center, “Dr. Félix Gómez”, Faculty of Medicine, University of Zulia, Maracaibo 4004, Zulia, Venezuela

## Abstract

The genetic, endocrine, and metabolic mechanisms underlying female reproduction are numerous and sophisticated, displaying complex functional evolution throughout a woman's lifetime. This vital course may be systematized in three subsequent stages: prenatal development of ovaries and germ cells up until* in utero* arrest of follicular growth and the ensuing interim suspension of gonadal function; onset of reproductive maturity through puberty, with reinitiation of both gonadal and adrenal activity; and adult functionality of the ovarian cycle which permits ovulation, a key event in female fertility, and dictates concurrent modifications in the endometrium and other ovarian hormone-sensitive tissues. Indeed, the ultimate goal of this physiologic progression is to achieve ovulation and offer an adequate environment for the installation of gestation, the consummation of female fertility. Strict regulation of these processes is important, as disruptions at any point in this evolution may equate a myriad of endocrine-metabolic disturbances for women and adverse consequences on offspring both during pregnancy and postpartum. This review offers a summary of pivotal aspects concerning the physiologic course of female reproductive function.

## 1. Introduction

Historically, the phenomenon of human reproduction has awakened great interest. One of its first scientific descriptions, authored by Hippocrates, dates back to the fifth century BC, suggesting generation of new beings to stem from the union of the male's ejaculate and the female's menstrual bleeding. More than two millennia after, we now know that reproduction derives from a complex succession of biologic events, where the union of the gametes, spermatozoa and oocytes, plays a fundamental role [1].

In their earliest stage, gametes originate from specific cells that abandon their somatic lineage to differentiate into primordial germ cells (PGC), key components in reproduction [2]. In female humans, the ovary represents an essential structural support for the development of PGC throughout their evolution [3]. Once they have matured into primordial follicles, a stage reached before birth, and once the subject has reached puberty, folliculogenesis begins, a series of cellular changes necessary for maturation and preparation for a second wave of structural and functional modifications inherent to the ovarian cycle, which in turn finalizes with the crucial event in female fertility: ovulation [4]. In ensemble, these processes permit generation of new life, reproduction.

Indeed, female reproductive physiology entails intricate interactions among hormonal, metabolic-energetic, genetic-epigenetic, and intra- and extraovarian factors, which in coordination modulate the successive development of the female gamete [5]. Disruptions in any of these components may lead to infertility, an alarming problem in women's global health, currently affecting 48.5 million females aged 20–44 years [6]. Moreover, alterations of female reproductive physiology often bear implications in other organ systems, as in the classical example of polycystic ovary syndrome [7]. Beyond the physical and mental implications in women [8], these alterations may also reflect on the ulterior health of their potential offspring [9]. Due to this profound impact in female well-being and their progeny, this review aims to describe the physiological and molecular phenomena implicated in female fertility.

## 2. Overview of Female Reproductive Function: Fertility as a Three-Act Theatre Piece

**Figure 1:**
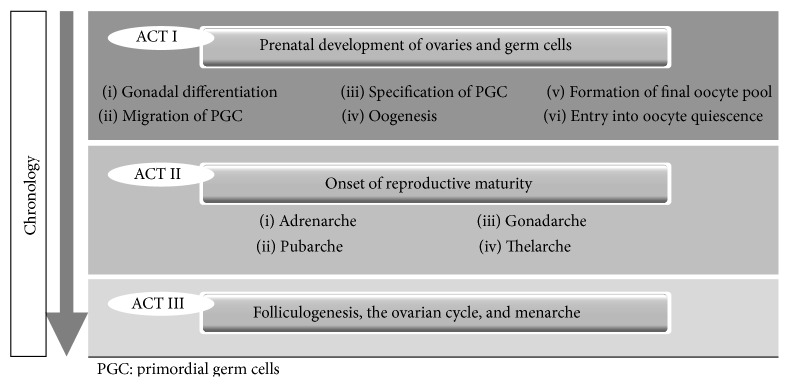
Main events in the physiologic life course of female reproductive function.

The ovary goes through a wide array of structural and functional modifications throughout a female's life, in order to provide reproductive potential [4].  Figure 1 chronologically summarizes the principal events in this timeline. These processes are subject to regulation by multiple endocrine signals, as reflected in the ample fluctuations in gonadotropin, sex hormone, and other mediators' serum concentrations throughout various stages of life (Table 1). Nonetheless, these molecules are only selected representatives from the abundance of mediators from many interconnected and overlapping neuroendocrine regulation systems, where both reproductive and metabolic signals are integrated [10].

Much like a theatrical play, this sequence may be schematized in three elemental parts or “acts”: (1) the setup: embryonic origin and* in utero* development of the ovary, and infantile quiescence; (2) the buildup: ovarian and adrenal reactivation in puberty and neuroendocrine cues initiating sexual maturation; and (3) the climax: molecular mechanisms in folliculogenesis and the normal ovarian cycle.

## 3. Act I: The Setup—Prenatal Development of Ovaries and Germ Cells

**Figure 2:**
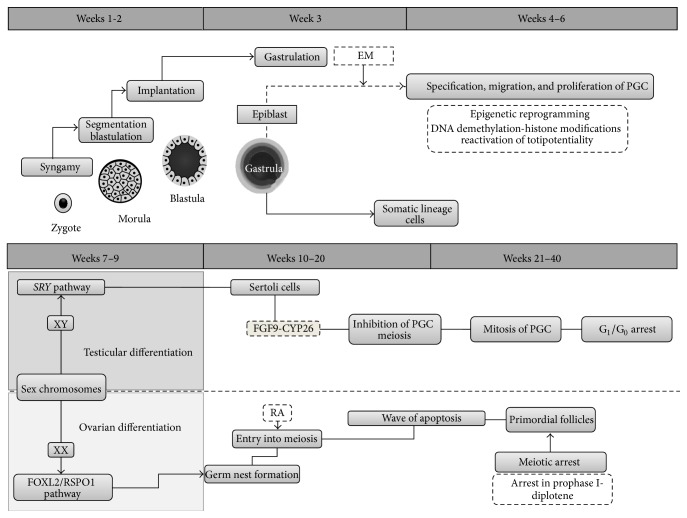
Overview of gonadal differentiation and germ cell development. CYP26: retinoic acid hydroxylases; EM: extraembryonic mesoderm; FGF9: fibroblast growth factor-9; PGC: primordial germ cells. RA: retinoic acid. Syngamy yields a single totipotent cell, the zygote, which subsequently undergoes several proliferative and reorganizational processes. After* gastrulation,* arrangement into a three-layered embryonic structure, has occurred, nascent PGC undergo induction into pluripotent cells,* specification,* by EM. PGC also begin* migration* towards their final residence, the gonadal ridges, whilst simultaneously suffering epigenetic reprogramming essential for reactivation of totipotentiality. Afterwards, according to the sex chromosome load, both PGC and gonads undergo* differentiation*. In XY subjects,* SRY* induces differentiation of Sertoli cells, which thereafter drive testicular differentiation. Likewise, FGF9 and RA hydroxylases inhibit meiosis in these PGC, instead favoring mitosis and then cell cycle arrest until puberty. In contrast, in XX individuals it is FOXL2/RSPO1 signaling in ovarian primordia that drives development of female gonads and germ nest formation. RA then induces entry into meiosis and proliferation. These cells later suffer a wave of apoptosis which determines the final pool of primordial follicles, which remain arrested in meiosis I until puberty.

In mammals, the prenatal period is a critical stage for the functional development of all organ systems. Within the female reproductive sphere, it comprises sexual differentiation, according to the chromosomal load inherited in syngamy, and formation of the future female gametes (Figure 2), both coordinated by successive genetic interactions [11].

### 3.1. Gonadal Differentiation

Gametes, spermatozoa and secondary oocytes, are haploid cells responsible for generation of offspring through fecundation, which culminates in the formation of a single cell, the zygote, whose genome proceeds from the conjugation of its predecessors' genomes. From this pivotal step, zygotes inherit a pair of sex chromosomes (XX or XY) which will drive the transformation of this cellular unit into a multicellular organism with a sex-specific phenotype [12]. Once the embryo has attained the definitive organization of germ layers, the urogenital ridge, a thickening of coelomic epithelium superimposed on the anterior portion of the mesonephros, becomes the gonadal primordium and the chief site for PGC development [13]. In beings with XY sex chromosome load, the* SRY* transcription factor binds to a promoter of* Sox9*, the Testis-Specific Enhancer of Sox9 Core Element (TESCO), which drives differentiation of Sertoli cells and in turn propels testicular formation. In absence of a Y chromosome, the undifferentiated or bipotential gonad does not express this gene, nor are its downstream mechanisms triggered, leading to formation of an ovary, the so-called standard process [14].

Although this description reflects the traditional approach to these phenomena as a “standard” or passive process, it is currently known to be a complex, active chain of events involving coordinated expression of a myriad of genes [15, 16]. Among these, the* FOXL2* transcription factor appears to play an important role by antagonizing* Sox9* activity [17]. Additionally, ovarian differentiation pathways involve R-spondin homolog 1 (RSPO1), a protein secreted in gonadal primordia increasing Wnt4 signaling which regulates *β*-catenin activity. This mediator can then translocate to the nucleus and interact with Hepatocyte Nuclear Factor 1 Homeobox A (HNF1A) to regulate transcriptional activity and cell adhesion in the ovary in formation [18].

### 3.2. Specification and Migration of Primordial Germ Cells

**Figure 3:**
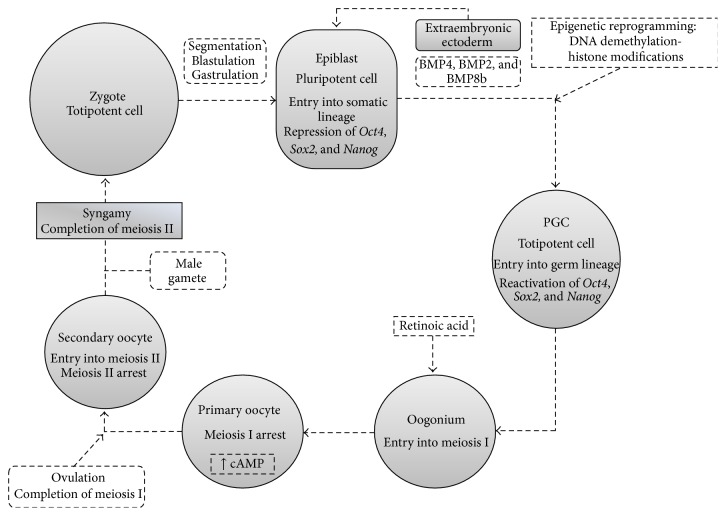
Cellular stages and signaling in female germ cell development. Syngamy produces a single totipotent cell, the zygote, which evolves through various structural stages. After gastrulation, extraembryonic ectoderm induces some epiblastic cells into pluripotency and entry into somatic lineage. These later undergo epigenetic reprogramming to regain totipotentiality and become primordial germ cells. These migrate to the gonadal ridges and suffer intense mitosis, becoming oogonia, which are then induced by retinoic acid to enter meiosis I. Primary oocytes are then arrested in this division and appear to survive until ovulation due to elevated intracellular cAMP levels. With ovulation, meiosis I is completed and meiosis II begins in secondary oocytes. Nonetheless, this division is only fulfilled if union with a male gamete occurs, which ultimately leads to formation of a zygote.

**Figure 4:**
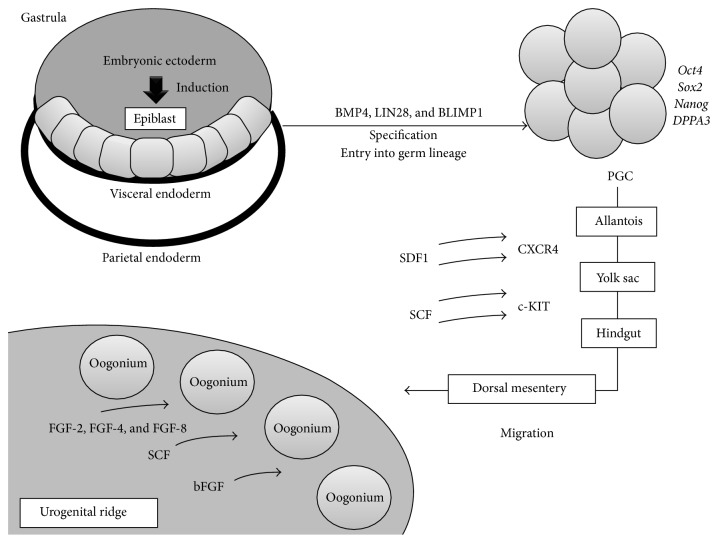
Factors controlling specification and migration of primordial germ cells. bFGF: basic fibroblast growth factor; FGF: fibroblast growth factor; PGC: primordial germ cells; SCF: Stem Cell Factor; SDF1: stromal cell-derived factor. Formation of fully competent gonads demands the presence of PGC in the genital primordia, which in turn requires the fulfillment of two fundamental processes. (1)* Specification*: PGC stem from a small group of cells which are subjected to induction by the extraembryonic ectoderm, via intense BMP4 signaling, which induces expression of BLIMP1 in these cells. The main factors controlling specification are the expression of pluripotent genes, for example,* Oct4*,* Sox2*, and* Nanog*, and thorough epigenetic reprogramming. In addition, BLIMP1 and LIN28 allow expression of DPPA3 (Stella), which mediates protection of maternal imprinting in PGC. (2)* Migration*: PGC initially reside with the epiblast in the gastrula in the posterior end of the primitive streak, which will later become the extraembryonic mesoderm. Then, PGC migrate through the allantois and reside temporarily in the yolk sac. These cells then migrate caudally through the hindgut towards the dorsal mesentery and then the urogenital ridges, their definitive location. The principal factors regulating this process are SDF1 and SCF, which bind to CXCR4 and c-KIT, respectively, mediating chemotaxis and survival of PGC.

PGC experience an extensive and complex succession of cellular transformations in order to become viable gametes ready for fecundation (Figure 3). Among embryonic cells, those who eventually evolve into PGC appear very early and must transition from the somatic to the germ lineage and reactivate their totipotentiality by effacing their progenitor imprinting, which constitute* specification* [19]. These processes require intense genetic and epigenetic reprogramming which happens simultaneously to these cells'* migration* towards the urogenital ridge (Figure 4) [20].

Circa day 6 after fecundation in mice, approximately the fourth-fifth week in humans [21], extraembryonic ectoderm subjects a small quantity of epiblastic cells from the primitive embryonic ectoderm to high levels of Bone Morphogenetic Protein 4 (BMP4) [22]. This induces expression of the* Prdm1* gene (PRD1-BF1 and RIZ domain-1) which codes for the B Lymphocyte-Induced Maturation Protein 1 (BLIMP1), which appears to be an important “switch” in transition to the germ lineage [23, 24]. LIN28 cooperates in this process by inhibiting Let7, a* Prdm1* repressor [25], and allowing expression of Stella, an early marker of PGC [26]. Loss of expression of various genes related to somatic development is also seen, such as* evx1*,* tbx1,* and* mesp1*, which are associated with development of ectodermic and mesodermic structures [27].

Roughly in day 8.5, between the fifth and eighth week in humans [21], these nascent PGC begin expressing genes typical of pluripotent cells such as* Oct4*,* Nanog*, and* Sox2* [28] and begin their migration towards the gonadal primordia [29]. Specific genetic programming coordinates the proliferation, survival, and migration of these cells. Molecules related to PGC migration include chemoattractants such as Stromal-Derived Factor (SDF1/CXCL12) [30] and Stem Cell Factor/c-Kit Ligand (SCF/KITL) [31], which bind to specific receptors expressed in the surface of PGC [32, 33]. Wnt3A is a glycoprotein which also appears to intervene in PGC migration and proliferation, presumably through stabilization of *β*-catenin [34].

Another molecular determinant of pluripotentiality in nascent PGC is expression of DNMT3 and DNMT4, families of DNA methyltransferases which mediate methylation of cytosine mainly in “CpG” (Cytosine-phosphate-Guanine) sequences, originating 5-methylcytosine (5mC) sites important for genomic imprinting [35]. These genomic stamps must be erased to achieve the totipotentiality inherent to fully specified PGC [36]. This demethylation is an active phenomenon that occurs around day 11.5 in mice, sixth-seventh week in humans [21], where TET1, TET2, and TET3, enzymes from the Methylcytosine Dioxygenase Ten-Eleven Translocation (TET) family, catalyze this process with oxidation of 5mC through iron- and 2-oxoglutarate-dependent mechanisms [37, 38].

Notably, these epigenetic changes do not affect maternal-origin genome in the embryoblast; therefore, methylation of various genes is maintained, generating epigenetic asymmetry in these loci in comparison to the paternal-origin genome [39]. The Developmental Pluripotency-Associated Protein 3 (DPPA3) or Stella factor, a 159-amino acid protein expressed in preimplantation embryos, embryonic stem cells, and PGC, appears to be the “molecular shield” of these loci. Stella is able to block TET3 activity on 5mC of the maternal-origin genome, and some paternal loci [39]. This selectivity derives from differential recognition of histones. The maternal genome is predominantly associated with demethylated H3 histones (H3K9me2), which are Stella binding targets [40]. This union appears to cause conformational disposition which impede TET3 activity on 5mC [38]. In consequence, Stella possesses a preservative effect on PGC imprinting [41].

In parallel, nascent PGC undergo histone modifications, presumably a mass substitution of histones mediated by HIRA and NAP1, as a sort of “repairing” required for restitution of totipotentiality [28]. Completion of this genetic-epigenetic reprogramming and localization of PGC in gonadal primordia mark the end of the specification and migration processes, respectively [11], which happen approximately in the sixth-seventh weeks in humans [42]. Cells that do not complete or deviate from either process suffer apoptosis due to a lack of prosurvival signals [43].

### 3.3. Oogenesis: Mitosis, Meiosis, and Meiotic Arrest

Once the genome has been reorganized, PGC continue abundant mitosis in gonadal primordia, leading to production of a great amount of cells, now denominated gonocytes, or oogonia in females, a process known as* oogenesis*. Sexual identity of gonocytes depends heavily on signals from their microenvironment. At this point, gonocyte expression of DAZL, an ARN-binding protein, is necessary not only to silence somatic genetic programming associated with markers of pluripotency, but also to facilitate an adequate response to these microenvironmental cues in the gonadal primordia [44, 45]. In this respect, the main messengers in ovarian primordia are SCF/KITL [46], basic fibroblast growth factor (bFGF), and fibroblast growth factors 2, 4, and 8 [47, 48]. In this scenario, around weeks 10–20 oogonia form cysts or transitory germ cell nests or clusters derived from multiple mitotic divisions that do not fully complete cytokinesis [49]. These nests are masses of approximately 16 germ cells which are interconnected by cytoplasmic bridges and enveloped by somatic cells that appear to be essential for the functional integrity of gametes [50].

Nevertheless, the fundamental event in oogenesis is induction of meiosis, destined to produce a haploid genome, necessary for syngamy [51]. This process begins roughly in weeks 11–13 in humans [52], with adjacent mesonephros-secreted retinoic acid (RA) as a key trigger [53]. Indeed, this molecule induces expression of the Stimulated by Retinoic Acid 8 (Strat8) protein in premeiotic germ cells, mediated by RXR nuclear receptors [54, 55]. Despite detailed downstream mechanisms remaining unclear, Strat8 signaling appears to downregulate synthesis of* Nanos2*, an inhibitor of meiosis through posttranscriptional modification of key mediators in this process [56, 57]. In addition, DAZL may exert a permissive role for RA signaling [58]. Epigenetic reprogramming appears to be a required step for initiation of meiosis, as both PGC and oogonia are subjected to high RA concentrations in the gonadal interstitium, yet only the latter enter meiosis [2].

In contrast, RA also participates in male sexual development, yet its effects are not seen until puberty, with male germ cells remaining arrested in G_0_/G_1_ until this stage, when meiosis is favored in this gender [55]. To this end, during* in utero* life and throughout infancy, RA is degraded by hydroxylases, CYP26B1 and CYP26C1, expressed in Sertoli cells [59]. Secretion of fibroblast growth factor-9 by Sertoli cells also contributes to inhibition of meiosis by upregulation* Nanos2* [57, 60], and by aiding in differentiation of Leydig cells [61].

In females, formation of germ cell nests and induction of meiosis occur simultaneously. By week 20, these clusters begin rupturing as some cells die through apoptosis, destabilizing the cystic structure. At this point, outlying somatic cells begin invading the nest and surround oocytes, defining the structure of primordial follicles [62, 63]. On the other hand, meiotic division encompasses two successive cycles, meiosis I and meiosis II, each with four phases: Prophase, Metaphase, Anaphase, and Telophase [64]. Oocytes advance through Prophase I and are arrested at the diplotene, where they remain quiescent awaiting induction of gonadotropin-dependent maturation at puberty, when they will obtain primary oocyte status [65]. This state is maintained by the Maturation Promoting Factor (MPF) complex, which consists of a catalytic subunit, the cyclin-dependent kinase 1 (CDK1), and a regulatory subunit, cyclin B1 (CB1) [66, 67]. CDK1 exhibits two regulatory sites that may be phosphorylated by WEE1/MYT1, inhibiting its activity [68, 69], whereas dephosphorylation by CDC25A permits progress through meiosis I [70]. This is facilitated by the constantly elevated levels of cAMP present in oocytes, which allow activation of Protein Kinase A (PKA), leading to reinforcement of WEE1/MYT1 and inhibition of CDC25A [69]. These high cAMP concentrations appear to originate from a constitutively activated stimulatory G protein-coupled receptor which supplies requirements of this second messenger [71]. Oocytes arrested in this phase may survive for years owing to their decondensed chromatin which facilitates gene transcription, as well as bidirectional communication with the surrounding somatic cells, which provide nourishment [72].

### 3.4. Programmed Cell Death: Formation of the Definitive Oocyte Pool

Two-thirds of all primordial oocytes suffer programmed cell death, in a phenomenon denominated “apoptotic wave,” considered a cellular “quality control” mechanism [73]. This is presumed to occur through the intrinsic apoptosis pathway, activated by two potential triggers: (a) suppression of prosurvival signals for oocytes [74] and (b) chromosomal alterations stemming from flaws in Prophase I [73]. The main prosurvival messengers are SCF/KITL [75], Leukemia Inhibitory Factor [76], and insulin-like growth factor I (IGF-I) [77], which induce expression of apoptosis modulatory proteins Bcl-XL, Bcl-2, and Bcl-w [78]. Autophagy may play a secondary role as another form of cell death in this context, although the causal correlation for each kind of death remains unknown [79].

Cells that survive the apoptotic wave constitute the final pool of primordial follicles available for the entirety of the female's reproductive life [80, 81]. Primary oocytes remain quiescent until puberty, when, with each iteration of the ovarian cycle, a luteinizing hormone (LH) surge will resume meiosis [82]. Indeed, female humans are born with approximately 1-2 million primordial follicles [83]. This reserve gradually wanes throughout the female's lifetime, as follicles abandon this pool due to either death or entry into folliculogenesis [84].

## 4. Act II: The Buildup—Onset of Reproductive Maturity

Puberty is the process through which male and female children become young adults, comprehending several events: (a) maturation of gametogenesis; (b) adrenarche, the onset of adrenal androgen synthesis and secretion; (c) pubarche, the appearance of pubic hair; (d) gonadarche, maturation of the hypothalamus-hypophysis-gonadal axis (HHGA), with gonadal sexual steroid synthesis and secretion; and, exclusively in females, (e) thelarche, onset of mammary development and (f) menarche, onset of ovulation and menstrual bleeding. Although the chronological order of these phenomena is widely variable, in conjunction, they allow for acquisition of full reproductive potential [85, 86].

The age at which each of these events occurs is highly variable and subject to a myriad of environmental and genetic factors [87]. For example, the National Health and Nutrition Examination Survey (NHANES III) found Caucasian girls to experience thelarche at a mean age of 10.4 years, whereas in their African American peers, the mean was 9.5 years [88]. Likewise, it tends to happen earlier in Mexican American girls and latest among Asian Americans [89]. These distinct patterns are also seen worldwide amongst different ethnical backgrounds [87, 90]. Nonetheless, most frequently, thelarche tends to be followed by pubarche, roughly 1–1.5 years afterwards, and menarche usually follows approximately 2.5 years after thelarche [91].

In recent years, onset of puberty appears to have transitioned to younger ages by 1-2 years; hypotheses explaining this shift encompass both extrinsic and intrinsic factors [92]. Regarding the former, endocrine-disrupting chemicals with estrogenic and antiestrogenic activity, such as polybrominated biphenyls and dichlorodiphenyltrichloroethane (DDT), are known to be powerful inductors of precocious puberty [93]. On the other hand, nutritional status appears to be the paramount intrinsic regulator of puberty onset parallel to genetic factors: girls with higher body mass index tend to display thelarche, pubarche, and even menarche much earlier, between 8 and 9.5 years of age; and conversely, low weight and malnutrition can significantly delay this process [94]. The following sections describe the molecular principles dictating the onset of puberty and its individual components.

### 4.1. Neuroendocrine Regulation of Sex Hormone Synthesis throughout Life

Although the HHGA is essential in reproductive function, it is active in stages as early as fetal development. Indeed, the fetal testicle begins functioning during the first half of gestation, firstly driven by human chorionic gonadotropin (hCG) and then by LH stimulation following the development of the hypothalamus-hypophysis portal system around weeks 11-12, with testicular androgens being key for male sexual differentiation [95]. In contrast, though the fetal ovary displays scarce steroidogenic activity with CYP11A1 and CYP17A1, this organ is considered to be functionally quiescent regarding hormone synthesis throughout* in utero* life and infancy, only accomplishing significant estrogenic synthesis at puberty; nevertheless, mechanisms underlying this latency remain unknown [96].

However, both genders appear to undergo a process described as the “newborn's miniature puberty,” a significant surge in HHGA activity after birth, presumably due to the relieving of GnRH secretion inhibition by maternal estrogen. This peak persists for roughly 12 months in females, where it manifests as moderate mammary development, and approximately 6 months in males, where it entails hyperplasia of Leydig and Sertoli cells, and a modest increase in size of external genital organs [91, 97].

This occurrence is succeeded by the “juvenile pause,” where secretion of GnRH, and consequently gonadal steroids, returns to quiescence as a result of full development of neural structures regulating this hypothalamic center [85]. This scenario underlines the pivotal role of GnRH pulsatile secretion as a “master switch” for maturation of the HHGA. In turn, this secretion pattern is subject to modulation by interactions of both inhibitory and excitatory neuroendocrine and synaptic systems on GnRH-secreting cells, amidst numerous endogenous and exogenous environmental signals. Dominance of excitatory signals permits this pulsatile pattern, leading to maturation of gonadal steroidogenesis:* gonadarche* [85, 97].

Glutamate and *γ*-amino-butyric acid (GABA) are the key excitatory and inhibitory hypothalamic neurotransmitters regarding puberty onset, respectively. A decrease in GABAergic tone, with a corresponding rise in glutamatergic tone, appears to be the fundamental process in this scenario [98]. The underlying trigger to this shift may be the expression of estrogen and progesterone receptors in glutamatergic and GABAergic neurons, which are absent previous to puberty, yet the stimuli for this sensitization to sex hormones remain incompletely understood [99]. Proposed mechanisms include a regulatory role for allopregnanolone, which appears to modify glutamate and GABA secretion, as well as modulate NMDA and GABA receptor expression in hypothalamic neurons [100], and an inhibitory effect by endogenous opioids, as suggested by precocious puberty induced by administration of naloxone [101].

Decreased GABAergic tone has been observed to be accompanied by a critical increase in kisspeptin signaling [102]. Kisspeptin, a hypothalamic neuropeptide coded by the* Kiss1* gene, has long been known to be crucial in sexual development, with mutations of the* GPR54* gene, which codes its receptor, being associated with a loss of reproductive function in both humans and mice [103, 104]. Hypothalamic disposition of kisspeptinergic neurons varies by species [105]; in humans, they have been located in both the arcuate nucleus (AN) and anteroventral periventricular nucleus (AVPV), most densely in the former [106]. During puberty, kisspeptin and GPR54 expression is upregulated in both nuclei, accompanied with greater GnRH secretion [107, 108]. In addition, kisspeptinergic signaling may be amplified by sex hormone-induced reorganization of neuronal projections in the hypothalamus [109].

Nonetheless, kisspeptinergic neurons in the AN and AVPV respond differently to sex steroid signaling: in the AVPV, sex hormones appear to favor LH secretion, completing a positive feedback circuit with the ovary that results in potentiation of gonadal steroid release, which may be especially important in the preovulatory LH wave [110]. On the other hand, stimulation of AN kisspeptinergic neurons appears to reduce LH secretion, suggesting a regulatory role [111].

These neuronal pathways are also subject to another level of regulation themselves: various signals reflective of the overall metabolic status are integrated into a modulatory “somatometer” [112]. These signals include leptin, glucose, and insulin levels, among many others [113]. Indeed, reproduction is an evolutionarily costly process in terms of energy expenditure and investment, and therefore, an optimal metabolic-energetic milieu is required for initiation of these phenomena [112].

Notoriously, leptin appears to mediate the impact of adipose depots on puberty onset. Leptin is a proteic adipokine secreted by both visceral and subcutaneous adipose tissue and participates not only in reproductive but also in immune and metabolic physiology [114]. Leptin may circulate freely or bind to its soluble receptor (sOB-R), which limits its availability for membrane receptors [115]. Approximately 40% of kisspeptinergic neurons in the AN express leptin membrane receptors [116], representing the fundamental link between adipose tissue and sexual development. In consonance, these elements act as sensors of energy storage, by facilitating GnRH pulsatile secretion in the presence of sufficient adipose tissue [117]. In addition, leptin also directly favors FSH and LH secretion [86]. Furthermore, expression of sOB-R appears to be inverse to adiposity and DHEAS levels, thus contributing to the role of obesity as an accelerator of puberty [118], and outlining a possible synergic mechanism between adrenarche and leptin for gonadarche induction.

Conversely, females with scarce body or intense physical activity often display disrupted GnRH secretion patterns [119]. Similarly, patients with anorexia nervosa frequently exhibit low gonadotropin and estradiol levels [120] associated with lower leptin concentrations in cerebrospinal fluid [121] and greater levels of circulating sOB-R [122]. Moreover, delay of puberty has been described as an adaptive mechanism in the face of scant energetic reserves, such as that seen in malnutrition and other conditions [123].

Notwithstanding this critical part of leptin in modulation of the HHGA, its role does not appear to be absolute, as leptin alone has been shown to fail to normalize LH secretion in animal models of caloric restriction-induced hypoleptinemia [124]. Thus, integration of other stimuli is also important. To this end, insulin appears to contribute by various pathways. Insulin appears to directly exert a positive, dose-dependent effect on GnRH secretion, as well as an inhibitory effect on GABAergic and neuropeptide Y-secreting neurons, both of which would suppress GnRH expression and secretion [125]. Insulin also has an indirect influence by regulating appetite in other hypothalamic centers [126].

Maturation of the HHGA is accompanied by the appearance of secondary sexual traits, propelled by sex steroids. In females, estrogens are key for the structural and functional development of the mammary glands. Estrogen receptor *α* is expressed in epithelial terminal ductolobular cells and is the main driver of mammary development during puberty, whereas the *β* isoform is found in myoepithelial cells, fibroblasts, and adipocytes in the breasts, yet it is considered to play a secondary role [127]. In breast tissue, estrogenic signaling appears to trigger paracrine and juxtacrine mediator secretion from epithelial cells, which in turn favor cell proliferation in neighboring cells [128].

Metabolic-energetic modulation is also implicated in breast development: both Growth Hormone (GH) and IGF-I, of both local and systemic origin, intervene much like estradiol, promoting expression of various growth factors [129], amongst which amphiregulin may be the most prominent as it amplifies all proliferative signals by potentiating local growth factor expression, permitting accelerated development of mammary glands during puberty [130].

### 4.2. Adrenarche and Pubarche: Role of Adrenal Androgens

*Adrenarche* entails maturation of the* zona reticularis* (ZR), the innermost layer of the adrenal cortex, accompanied by an increase in adrenal androgen synthesis and secretion, specifically 19-carbon DHEA and DHEAS, and apparition of androgen-dependent hair,* pubarche*, its fundamental clinical manifestation [131].

In contrast to the salient role adrenal androgens serve during fetal life, where they are key precursors for augmented estrogen synthesis during gestation [132], their significance concerning adult reproductive function remains unclear. Indeed, the onsets of adrenarche and gonadarche are largely independently regulated, and adrenarche does not appear to be necessary for gonadarche to take place [133], although premature maturation of the HHGA in subjects with congenital adrenal hyperplasia suggests the existence of a currently unelucidated link [134].

Likewise, the unequivocal mechanisms underlying the initiation of adrenarche are also unclear. Intrinsic and autonomous modifications in adrenal structure and function may be one of the key phenomena in this aspect: after birth, the fetal zone of the adrenal gland devolves, allowing for an expansion of the neocortex, with well-defined* zona fasciculata* and* zona glomerulosa*, yet scarce ZR-like cells [135]. This shift is associated with an acute decrease in DHEA and DHEAS synthesis. This adrenal architecture persists during infancy, with only sparse ZR-like islets until adrenarche, where the ZR acquires its adult configuration. Nonetheless, the molecular mechanisms underlying this timeline remain cryptic [136].

Regarding hormonal signals for adrenarche, ACTH is not considered a trigger as its serum levels do not change during this event, although it appears to play a permissive role, as individuals with ACTH receptor mutations fail to undergo adrenarche [137]. Alternative proteolytic derivatives of proopiomelanocortin, the precursor of ACTH, have also been proposed, yet results have been inconclusive [138]. Likewise, CRH may be able to prompt DHEA synthesis, but the relative impact of CRH versus ACTH activity in this scenario is undiscerned [139].

Finally, metabolic status may also be an important regulator of adrenarche. This influence appears to begin as early as* in utero*, where low birth weight might trigger adrenal hyperfunction at adrenarche, with reports of an inverse relationship between birth weight and DHEAS levels, independent of cortisol levels [140]. Similarly,* in vitro* treatment of fetal adrenal cells with insulin, IGF-I, and IGF-II has been linked with significant increases in DHEAS synthesis [140], as well as augmented sensitivity to ACTH signaling, with greater CYP17A1 and 3*β*HSD2 expression [141]. In addition, leptin may mediate the impact of obesity in this event: it has proved to increase CYP17A1 activity* in vitro* [142], although its significance* in vivo* during adrenarche is undetermined [143].

**Figure 5:**
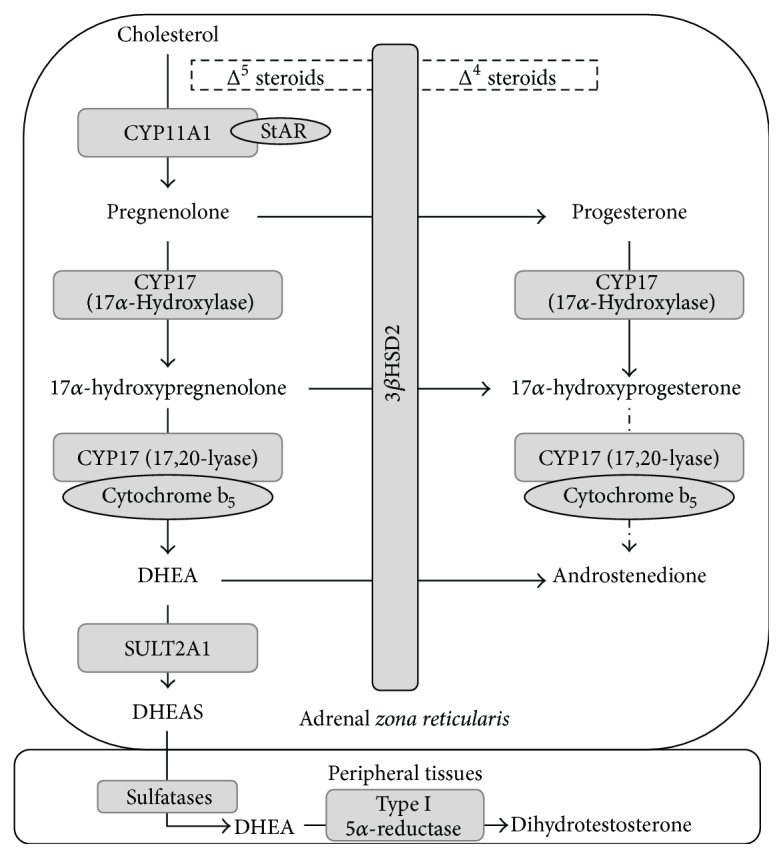
Steroidogenesis pathways and regulation in the adrenal* zona reticularis*. 3*β*HSD2: 3*β*-hydroxysteroid dehydrogenase 2; CYP11A1: cholesterol side-chain cleavage enzyme; CYP17: 17*α*-hydroxylase/17,20 lyase; DHEA: dehydroepiandrosterone; DHEAS: dehydroepiandrosterone sulfate; StAR: Acute Steroidogenic Regulatory Protein. After CYP11A1 acts on cholesterol, pregnenolone may undergo both functions of CYP17 (17*α*-hydroxylation and 17,20-lyation, the latter with cytochrome b_5_ as a cofactor), rendering DHEA as a product. DHEA synthesis is favored in the* zona reticularis* because the 17,20-lyase function acts preferentially on Δ^5^ steroids, whose production is potentiated in adrenarche due to 3*β*HSD2 underexpression. Additionally, upregulated SULT2A1 expression augments DHEA sulfonation, preventing conversion to other metabolites. DHEAS is secreted and reconverted to DHEA by sulfatases in peripheral tissues, where it also undergoes type 5*α*-reductase catalysis and then exerts its effects.

Regardless of the initiating signals, the fundamental characteristic of adrenarche is an increase in adrenal androgen levels (Figure 5). This augmentation stems from the coordinated interactions of CYP11A1 and CYP17A1 mainly, alongside 3*β*HSD2 and SULT2A1 [131]. Firstly, CYP11A1 acts in consonance with StAR signaling, both stimulated by ACTH, driving quantitative upregulation of adrenal steroidogenesis [144]. On the other hand, CYP17A1 exhibits dual function, exhibiting both 17*α*-hydroxylase and 17,20-lyase activity on 21-carbon steroids, which leads to formation of 19-carbon molecules, such as DHEA [145]. Whereas its 17*α*-hydroxylase activity displays comparable efficacy in both Δ^4^ and Δ^5^ steroids, its 17,20-lyase activity shows predilection for Δ^5^ substrates. Therefore, DHEA is the main product of this enzyme, with 17*α*-hydroxypregnenolone as an intermediary metabolite, from which DHEAS and androstenedione are obtained [146]. During adrenarche, the ZR displays increased expression of not only CYP17A1, but also cytochrome b_5_, a hemoprotein required for the 17,20-lyase function of CYP17A1. This cofactor is preferentially colocalized with CYP17A1 in the ZR, with lesser expression in other zones, partly explaining the absence of a significant increment in glucocorticoid synthesis during adrenarche [147].

Additionally, 3*β*HSD2 does not participate in the DHEA pathway but plays an indirect synergic role. This enzyme catalyzes the conversion of Δ^5^ steroids to their Δ^4^ homologues [145]. By unknown mechanisms, this enzyme is downregulated in the ZR during adrenarche, resulting in potentiated DHEA production down the Δ^5^ pathway [131]. Lastly, SULT2A1 is upregulated in adrenarche, thus assuring that metabolites continue down the pathway for Δ^5^ steroids, as sulfonation impedes activity by CYP17A1 and 3*β*HSD2, ultimately favoring DHEAS synthesis in the ZR [148].

Although DHEAS is biologically inactive, it may be reconverted to DHEA by sulfatases in peripheral tissues and subsequently converted to dihydrotestosterone, an active androgen, by 5*α*-reductase. This step is essential, as it exponentially multiplies its bioactivity in comparison to the weaker adrenal androgens [149]. The key manifestation of adrenarche is pubarche, the development of androgen-dependent hair in the pubic, axillar, and pectoral areas, as well as facial hair in males. Moreover, development of cutaneous apocrine glands originates a characteristic body odor [137].

## 5. Act III: The Climax—Folliculogenesis and the Ovarian Cycle

**Figure 6:**
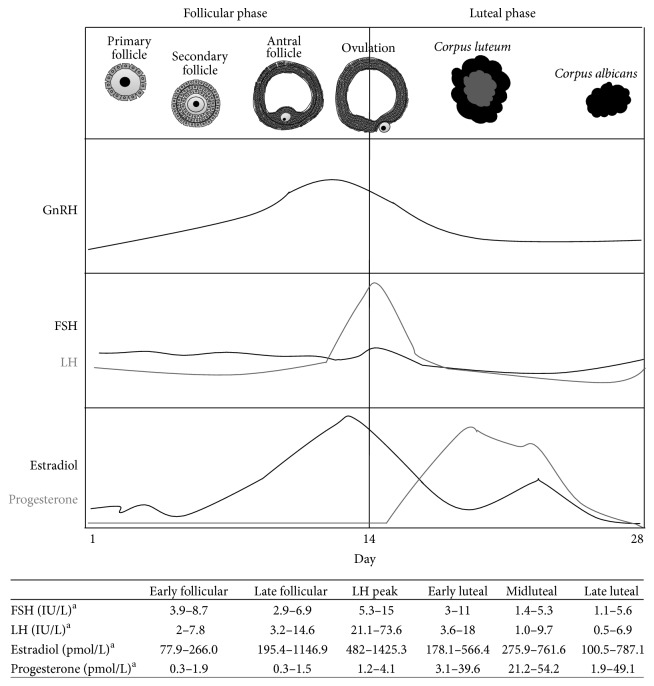
Evolution of the ovarian follicle and hormonal levels throughout the ovarian cycle. FSH: Follicle-Stimulating Hormone; GnRH: Gonadotropin-Releasing Hormone; LH: luteinizing hormone.  ^a^Stricker et al. [151]. The first day of the ovarian cycle, GnRH levels commence a progressive rise, which is accompanied by FSH and, to a lesser extent, LH secretion. Ovarian stimulation by gonadotropins leads to a gradual increase in estradiol levels, which towards midcycle induce a LH peak. This LH acme triggers ovulation and thus begins formation of the* corpus luteum*. This tissue will then progressively achieve a peak in progesterone secretion and then gradually decline as gonadotropin levels drop and the* corpus luteum* degenerates into the* corpus albicans,* marking the end of the luteal phase, and the beginning of a new cycle.

Once histologic and functional maturity is attained by the components of the HHGA, the ovarian cycle begins, involving a series of endocrine interactions oriented to the expulsion of oocytes,* ovulation*, which, owing to parallel modifications in the endometrium, offer the necessary support for implantation, thus acting coordinately to assure female fertility [150, 151]. The ovarian cycle comprises two phases, follicular and luteal (Figure 6), each with distinct endocrine profiles hereby summarized.

### 5.1. The Follicular Phase: Preparing for Ovulation

Ovarian follicles are the fundamental morphophysiologic units of the ovaries, as they represent the main endocrine and reproductive compartment in this organ. Primordial follicles present at birth may either perish, as part of ovarian senescence, or enter folliculogenesis (FG) [152]. FG encompasses a succession of cell changes required for maturation of ovarian follicles, in preparation for ovulation [153].

**Figure 7:**
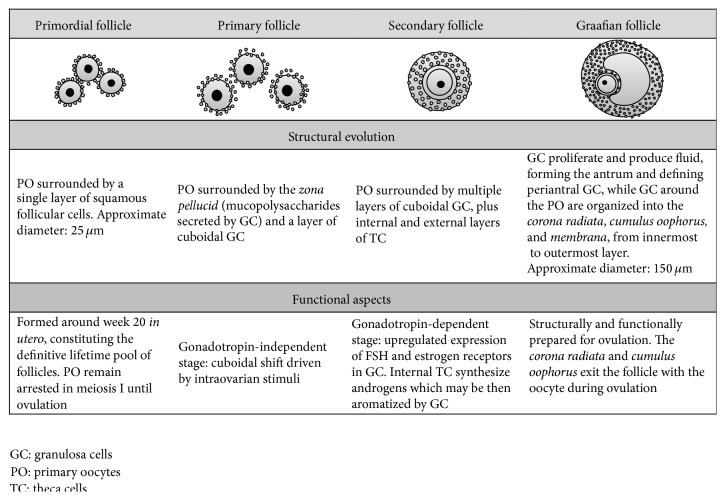
Follicular stages in folliculogenesis.

Starting from primordial follicles, that is, oocytes surrounded only by a monolayer of squamous granulosa cells (GC), this sequential development depicts 4 typical stages: primary, secondary, and tertiary or Graafian follicles [153] (Figure 7). The first structural shift in FG involves transformation of squamous GC into cuboidal cells, which define the primary follicle [154]. Afterwards, at least two layers of cuboidal GC exist in secondary follicles, which also exhibit upregulated FSH, estrogen, and androgen receptor expression [155], as well as an additional layer of somatic cells, theca cells (TC), in the external surface of the basal lamina [19]. The latter determines cell polarity and aids in control of proliferation and differentiation [156].

These early modifications appear to be FSH-independent and rely on intraovarian mechanisms [157]. Peptides such as bFGF, IGF-I, Epidermal Growth Factor, and Growth Differentiation Factor-9 (GDF-9) are important in this respect, as they are expressed by oocytes during FG, promoting differentiation and proliferation of GC, stimulating development of TC, inhibiting differentiation into luteocytes, and promoting estradiol secretion [158]. GDF-9 appears to be the principal driver of these effects until entry into the antral stage [159]. Likewise, anti-Müllerian hormone, a member of the Transforming Growth Factor-*β* (TGF-*β*) family, is a powerful inhibitor of follicular growth, governing entry of primordial follicles into FG [159].

Later events depend on FSH and, secondarily, LH signaling [160], including hyperplasia an hypertrophy of GC and TC, as well as the apparition of estrogen-rich fluid-filled spaces among GC, due to the osmotic gradient produced by the hyaluronan and chondroitin sulfate molecules present in GC, as well as upregulation of aquaporins and remodeling of intercellular junctions [161]. The additive effect of these changes facilitates a rapid increase in follicular volume and coalescence of these spaces, leading to formation of the antrum, which defines Graafian follicles [162].

Selection of a dominant follicle is paramount in order to preserve the integrity of the ovarian cycle [163]. Complex endocrine interplay underlies this aspect: around the middle of the follicular phase, the progressive increase in circulating FSH levels induces expression of LH receptors and aromatase in ovarian follicles [159]. In consequence, estradiol secretion by GC increases, leading to suppression of FSH secretion, marking a transition from FSH- to LH-dependent stimulation [164]. This shift hinges on the “rescue” of the dominant follicle from all other FSH-recruited follicles in development which will subsequently suffer atresia. The LH-rescued follicle is also prepared to respond to the LH peak later in the ovarian cycle [153].

Activins and inhibins are important regulators of dominant follicle growth [165]. These messengers belong to the TGF-*β* family [166] and as such are active as dimers: activins are constituted by two *β* subunits, *β*A, *β*B, *β*C, or *β*D; the most widely studied are activin A (a *β*A-*β*A homodimer), activin B (a *β*B-*β*B homodimer), and activin AB (a *β*A-Βb heterodimer) [167]. On the other hand, inhibins are comprised of an *α* subunit disulfide linked to one of the activins *β*, yielding two heterodimers: inhibin A (*α*-*β*A), or inhibin B (*α*-*β*B) [168]. Both activins and inhibins are synthesized in ovarian follicles, as well as hypophyseal gonadotropic cells and placental tissue, among others [169].

Activins directly intervene in follicular development in two principal manners: (a) extraovarian effects, by favoring FSH synthesis at the hypophysis, and (b) intraovarian autocrine signaling by GC, self-stimulating proliferation, and upregulation of aromatase and FSH receptor expression in these cells within the dominant follicle [170]. Preferential induction of aromatase expression by IGF-II in CG of the dominant follicle may play a secondary role in this scenario [171]. Once the antral stage is reached, activins also upregulate LH receptor expression in TC, essential for the LH-mediated rescue of the dominant follicle [172]. Additionally, activins appear to attenuate LH-induced androgen secretion in TC [173]. Activin signaling is regulated by follistatin, an autocrine monomeric glycoprotein also secreted by GC, which binds to activin and blocks its receptor-binding residues [174].

As the follicular phase transpires and the dominant follicle increases in size and estrogen synthesis, activin levels drop and inhibin levels rise [175]. Inhibins are also released by GC and are particularly relevant during the antral stage, as they enhance androgen production by TC, which are necessary substrates for subsequent aromatization [176]. Moreover, inhibins also seem to directly interfere with growth of all nondominant follicles [170, 173].

**Figure 8:**
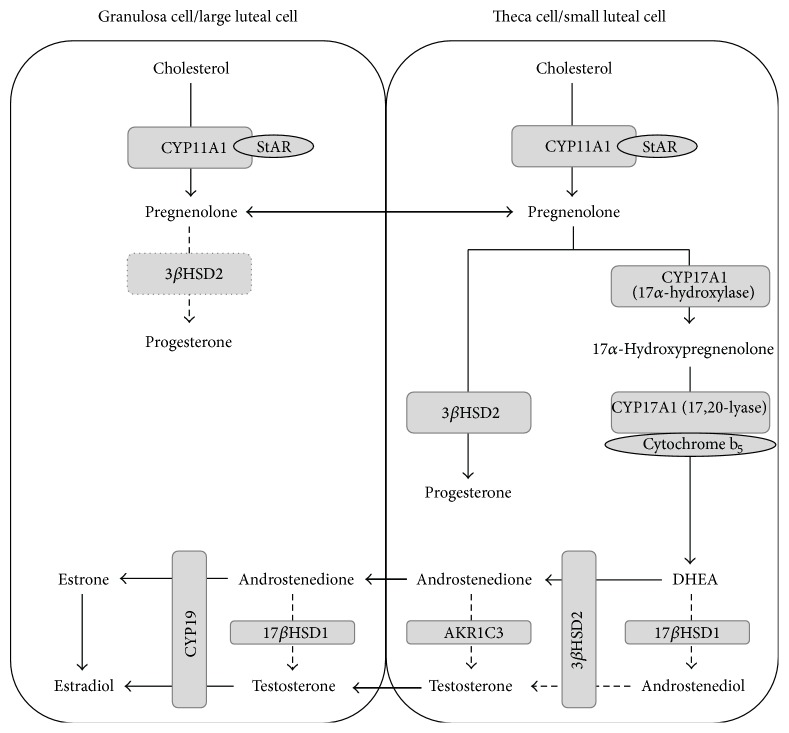
Ovarian steroidogenic pathways. Solid lines are major pathways. Dashed lines are minor pathways. Dotted line represents route only available after luteinization. Double lines represent intercellular steroid traffic. Underlined steroids are major secretion products. 3*β*HSD2: 3-*β*-hydroxysteroid dehydrogenase 2; 17*β*HSD1: 17-*β*-hydroxysteroid dehydrogenase 1; AKR1C3: aldo-keto reductase family 1 member C3; CYP11A1: cholesterol side-chain cleavage enzyme; CYP17A1: 17*α*-hydroxylase/17,20 lyase; CYP19: aromatase; DHEA: dehydroepiandrosterone; StAR: Acute Steroidogenic Regulatory Protein.

Because steroid hormones are the key hormonal products of the ovarian cycle with fundamental effects both systemically and in the ovary, ovarian steroidogenesis is tightly regulated [145]. Indeed, in ovarian follicles, steroid metabolism is compartmentalized between GC and TC during the follicular phase (Figure 8), both of which possess prominent smooth endoplasmic reticula and abundant lipid vesicles, typical features of steroidogenic cells [177]. GC express CYP11A1, CYP19, and 17*β*HSD1, while TC express CYP17A1 and scant levels of CYP11A1. Therefore, the preliminary product of GC is pregnenolone following cleavage of the cholesterol side-chain by CYP11A1, which diffuses to TC to be converted chiefly to androstenedione by CYP17A1. This androgen returns to GC to finalize the enzymatic pathway towards estradiol, the key steroid hormone product during the follicular phase [145].

Estrogens induce uterine modifications parallel to these ovarian events, inducing endometrial proliferation with intense mitotic activity in its epithelium and stroma, resulting in a near triplication of endometrial thickness, accompanied with elongation and coiling of spiral arteries [178, 179].

### 5.2. Ovulation: The Big Bang in Female Fertility

Shortly before midcycle, estrogen concentrations reach their peak, resulting in a LH wave critical for ovulation. This acme is achieved due to recruitment of a promoter region in* Kiss1* by estrogen receptor *α* isoforms in kisspeptinergic neurons in the AVPV, thus upregulating kisspeptin synthesis and secretion, which in turn boosts GnRH and LH secretion and ultimately raises ovarian estrogen secretion, thus completing a positive feedback circuit [180].

The resulting elevation in LH levels promotes progesterone secretion and augments plasminogen activator in GC [181, 182], leading to increased tissue plasmin which activates collagenases and stimulates TNF release by TC, thus enhancing collagenolysis by inducing expression of matrix metalloproteinases. The integrated effect of these mechanisms is the weakening of follicular walls on their apical side [183]. Additionally, TNF potentiates local prostaglandin synthesis [184]; and LH drives follicular angiogenesis and vascular remodeling, primarily through induction of vascular endothelial growth factors [185], resulting in plasma transudation to the inside of follicles, with follicular swelling. In consonance, follicular wall degeneration and swelling finalize in ovulation: follicular rupture, with expulsion of the oocyte and antral fluid [186].

Preceding ovulation, the oocyte must resume meiosis I and progress through meiosis II. Oocytes quiescent in Prophase I possess an intact nuclear envelope, in a stage known as germinal vesicle, whose rupture is an early sign of recommencement of meiosis [69]. Because the LH surge is associated with this breakdown, yet no LH receptors are present in oocytes, indirect mechanisms are suspected to convey this signaling from GC and TC to oocytes, yet remaining obscure [187]. Nonetheless, the key outcome is a decrease in intracellular cAMP, which may be due to (a) increased cGMP concentration with activation of phosphodiesterase 3A, thus favoring cAMP degradation [188]; (b) activation of inhibitory G proteins [189]; or (c) disruption of stimulatory G protein-coupled receptors [190]. Lower cAMP levels result in relief of PKA-mediated inactivation of CDC25B, which is then free to inhibit WEE1/MYT1 activity, thus allowing MPF to promote cell division [69]. This division is unequal, leading to formation of a polar body much smaller than the oocyte. Indeed, asymmetric spindle pole attachment leads to cortical activation of CDC42 during Anaphase, which determines the surface of forming polar body [191]. This process is aided by a RhoA-based contractile ring whose constriction contributes to localization of one spindle pole and one set of chromosomes into the CDC42 budding compartment [192].

Once meiosis I is completed, the oocyte immediately enters meiosis II, where cytostatic factor maintains MPF in a stable state, allowing inhibition of the Anaphase-promoting complex/cyclosome (APC/C) and therefore halting progress through the cell cycle in Metaphase II, thus preventing parthenogenetic activation and development of an embryo without paternal genomic contribution [193]. These characteristics define the secondary oocyte, whose cell cycle only continues with syngamy, when the spermatozoon triggers a calcium-calmodulin Protein Kinase II-mediated disinhibition of APC/C [193, 194]. This occurs due to proteosomal degradation of cyclin B 26S subunit, resuming meiosis II with transition from Metaphase to Anaphase [195].

### 5.3. The Luteal Phase: A Time Window for Implantation

After ovulation,* luteinization* occurs in the ovaries, a conglomerate of architectural and physiologic changes aimed to offer support for the newly released oocyte. Although these changes are intensified after ovulation, they begin approximately 36 hours before ovulation, driven by the LH surge typical of this time, which in turn obeys increased GnRH pulses [196]. Furthermore, preovulatory luteinization is essential for follicular rupture, as it entails induction of COX-2 expression in GC undergoing luteinization, with increased production of PGE2 [197]. In turn, this mediator promotes synthesis of tissue plasminogen activator (tPA), favoring fibrinolysis and oocyte release [198].

After follicular rupture, this tissue undergoes thorough reorganization, with formation of the* corpus luteum* and mitotic arrest of its constituent cells. Steroidogenic cells suffer phenotypical modifications: TC become small luteal cells (SLC); and GC become large luteal cells (LLC) [199]. SLC retain the androgenic synthesis capacity of TC, and LLC keep aromatase expression as seen in GC. However, LLC begin expressing 3*β*HSD2, allowing progesterone secretion from both cell types, although it is greater in LLC (Figure 8) [200]. Although in SLC progesterone synthesis is directly induced by LH, via PKA activation and StAR phosphorylation [201], in LLC it appears to depend on PGE2 for PKA activation [202]; yet LLC also seem to require lower levels of cAMP for this event in comparison to SLC [203]. Additionally, SLC appear to express 5*α*-reductase, 5*β*-reductase, and 3*α*-hydroxysteroid oxidoreductase, the key enzymes for allopregnanolone synthesis [204], a neurosteroid important for modulation of estrous behavior [205].

These rearrangements of steroidogenic cells are concomitant with extensive angiogenesis in order to offer nutrition to this tissue, driven by local neutrophil- and macrophage-secreted messengers [206]. These include VEGF-4, which acts through delta-like ligand-4/notch signaling [207], and nestin, a filamentous protein associated with* de novo* development of capillaries [208]. Nitric oxide is another fundamental regulator, which, depending on the ovarian microenvironment and under regulation of prostaglandin F2*α*, may act as either a luteotropic or luteolytic agent through modulation of angiogenesis [209].

These events lead to the elevated progesterone synthesis and secretion typical of this phase, which in turn is oriented to setting an optimal stage for implantation, thus maintaining the functional correlation between the ovarian uterine cycles [210]. These effects include augmented secretion of glycogen and mucus and greater tortuosity of spiral arteries [211]. Likewise, invasion by immune cells is increased, chiefly by NK cells, macrophages, and T cells, which reach their peak during this phase and are destined to regulate trophoblastic invasion and angiogenesis [212, 213].

Increased levels of sex hormones also constitute early signals for luteolysis, by lowering hypophyseal gonadotropin secretion through negative feedback [210]. At a cellular level, both the extrinsic, Fas/Fas-L-dependent [213], and intrinsic apoptosis pathways seem to be involved. Regarding the latter, luteocytes possess numerous prosurvival signals such as LH, leptin, and glucocorticoids, which suppress expression of intrinsic proapoptotic proteins such as Bax and cIAP-2 [214], whereas luteocyte stress can activate the p53 pathway [215]. Ultimately, luteolysis finalizes with hyalinization of the* corpus luteum* as luteocytes die, originating the* corpus albicans*. This process implicates synthesis of extracellular matrix by ovarian fibroblasts [216], whilst the eventual resorption of these remnants, and the consequent restitution of preovulatory ovarian architecture, depends on activity by local macrophages and myofibroblasts [217].

## 6. Resolution: Concluding Remarks

After ovulation, the course of female reproductive function pivots fundamentally on the presence of fecundation. Indeed, if absent, the* corpus luteum* will swiftly degenerate, prompting the reinitiation of the ovarian cycle and the beginning of menstrual bleeding. On the other hand, the presence of an adequately implanted zygote in the endometrium will prompt the decidual reaction, an increase in endometrial secretion and stromal edema [218]. These modifications allow for adequate syncytiotrophoblast development, which, in turn, is able to grant maintenance to the corpus luteum via hCG secretion, representing one of the first of many endocrine modifications inherent to gestation [219].

Under healthy conditions, the ovarian cycle periodically gives rise to this bifurcation until menopause, the natural cessation of the ovaries' primary function: folliculogenesis and the ovarian cycle [220]. Early stages of this transition feature briefer cycles, owing to shorter follicular phases with smaller-sized follicles [221]. This phenomenon appears to be due chiefly to a decrease in inhibin B and AMH synthesis, which leads to augmented FSH release and thus increased estrogen synthesis. In turn, this would facilitate earlier triggering of the LH surge [222]. Secretion patterns of the latter and GnRH are also altered, with a decline in pulse frequency [223], in association with disruptions of the neural networks modulating GnRH release [224]. In consonance with reduced signaling by inhibin B and AMH, increased expression of proapoptotic genes in oocytes propels accelerated depletion of follicle reserve until its eventual exhaustion [225]. In this scenario, there is a significant decline in circulating estradiol levels, as only extraovarian sources of this hormone remain active, in particular, adipose tissue [226]. Lower estrogen levels entail a broad range of multisystemic changes in physiology [220]. Indeed, menopause, the “curtain call” of female reproductive function, is a well-recognized risk factor for cardiovascular disease and osteoporosis, among many other disorders [227].

Further understanding of the molecular mechanisms underlying female fertility is required in order to provide better management to the multiple disturbances which may occur within its complex regulatory systems, as these have consequences on global female health beyond the reproductive sphere.

## Figures and Tables

**Table 1:** Approximate values for serum levels of various endocrine mediators at distinct stages in the physiologic life course of female reproductive function.

Stage	LH (IU/L)^a^	FSH (IU/L)^a^	Estradiol (pg/mL)^a^	DHEAS (*μ*g/dL)^a^	Testosterone (ng/dL)^a^	Leptin (ng/mL)	IGF-I (*μ*g/dL)
Prepubertal	>0.3	<4	<10	5–40	<20	0.5–11.7^b^	115–208^d^
Premenarchal	≤12	1–12	<50	35–130	13–44	1.6–12.6^c^	232–363^e^
Postmenarchal (early follicular phase)	2–11	1–12	20–85	75–255	15–59	5.8–19.2^c^	251–440^e^
Postmenarchal (midcycle)	≤85	≤19	≤350	—	—	—	—

LH: luteinizing hormones; FSH: Follicle-Stimulating Hormone; DHEAS: dehydroepiandrosterone sulfate.

^a^Bordini and Rosenfield [91].

^b^Ellis and Nicolson [228].

^c^Bandini et al. [229].

^d^Yüksel et al. [230].

^e^Zumbado et al. [10].

## Abbreviations

17*β*HSD1: 17-*β*-Hydroxysteroid dehydrogenase 1
3*β*HSD2: 3-*β*-Hydroxysteroid dehydrogenase 2
5mC: 5-Methylcytosine
ACTH: Adrenocorticotropic hormone
AN: Arcuate nucleus
AVPV: Anteroventral periventricular nucleus
bFGF: Basic fibroblast growth factor
cAMP: Cyclic adenosine monophosphate
CRH: Corticotropin-releasing hormone
CYP11A1: Cholesterol side-chain cleavage enzyme
CYP17A1: 17*α*-Hydroxylase/17,20-lyase
CYP19: Aromatase
DHEA: Dehydroepiandrosterone
DHEAS: Dehydroepiandrosterone sulfate
FG: Folliculogenesis
FSH: Follicle-Stimulating Hormone
GABA: *γ*-Amino-butyric acid
GC: Granulosa cells
GH: Growth Hormone
GnRH: Gonadotropin-Releasing Hormone
hCG: Human chorionic gonadotropin
HHGA: Hypothalamus-hypophysis-gonadal axis
IGF-I: Insulin-like growth factor I
IGF-II: Insulin-like growth factor II
LH: Luteinizing hormone
LLC: Large luteal cells
MPF: Maturation Promoting Factor
PGC: Primordial germ cells
PKA: Protein Kinase A
RA: Retinoic acid
SLC: Small luteal cells
SULT2A1: DHEA sulfotransferase
SCF/KITL: Stem Cell Factor/c-Kit Ligand
StAR: Acute Steroidogenic Regulatory Protein
TC: Theca cells
ZR: Zona reticularis.

## References

[1] Yanagimachi R. (2012). Fertilization studies and assisted fertilization in mammals: their development and future. *Journal of Reproduction and Development*.

[2] Spiller C. M., Bowles J., Koopman P. (2012). Regulation of germ cell meiosis in the fetal ovary. *International Journal of Developmental Biology*.

[3] Toloubeydokhti T., Bukulmez O., Chegini N. (2008). Potential regulatory functions of MicroRNAs in the ovary. *Seminars in Reproductive Medicine*.

[4] Baerwald A. R., Adams G. P., Pierson R. A. (2012). Ovarian antral folliculogenesis during the human menstrual cycle: a review. *Human Reproduction Update*.

[5] Dumesic D. A., Abbott D. H. (2008). Implications of polycystic ovary syndrome on oocyte development. *Seminars in Reproductive Medicine*.

[6] Mascarenhas M. N., Flaxman S. R., Boerma T., Vanderpoel S., Stevens G. A. (2012). National, regional, and global trends in infertility prevalence since 1990: a systematic analysis of 277 health surveys. *PLoS Medicine*.

[7] Rojas J., Chávez M., Olivar L. (2014). Polycystic ovary syndrome, insulin resistance, and obesity: navigating the pathophysiologic labyrinth. *International Journal of Reproductive Medicine*.

[8] Souter V. L., Hopton J. L., Penney G. C., Templeton A. A. (2002). Survey of psychological health in women with infertility. *Journal of Psychosomatic Obstetrics and Gynecology*.

[9] Dupont C., Armant D. R., Brenner C. A. (2009). Epigenetics: definition, mechanisms and clinical perspective. *Seminars in Reproductive Medicine*.

[10] Zumbado M., Luzardo O. P., Lara P. C. (2010). Insulin-like growth factor-I (IGF-I) serum concentrations in healthy children and adolescents: Relationship to level of contamination by DDT-derivative pesticides. *Growth Hormone and IGF Research*.

[11] Hodgen G. D. (1989). Neuroendocrinology of the normal menstrual cycle. *The Journal of Reproductive Medicine*.

[12] Wilhelm D., Palmer S., Koopman P. (2007). Sex determination and gonadal development in mammals. *Physiological Reviews*.

[13] Ikawa M., Inoue N., Benham A. M., Okabe M. (2010). Fertilization: a sperm's journey to and interaction with the oocyte. *The Journal of Clinical Investigation*.

[14] Liu C.-F., Liu C., Yao H. H.-C. (2010). Building pathways for ovary organogenesis in the mouse embryo. *Current Topics in Developmental Biology*.

[15] Piprek R. P. (2010). Molecular and cellular machinery of gonadal differentiation in mammals. *International Journal of Developmental Biology*.

[16] Piprek R. P. (2009). Molecular mechanisms underlying female sex determination—antagonism between female and male pathway. *Folia Biologica*.

[17] Biason-Lauber A. (2012). WNT4, RSPO1, and FOXL2 in sex development. *Seminars in Reproductive Medicine*.

[18] Schmidt D., Ovitt C. E., Anlag K. (2004). The murine winged-helix transcription factor Foxl2 is required for granulosa cell differentiation and ovary maintenance. *Development*.

[19] Edson M. A., Nagaraja A. K., Matzuk M. M. (2009). The mammalian ovary from genesis to revelation. *Endocrine Reviews*.

[20] Seisenberger S., Andrews S., Krueger F. (2012). The dynamics of genome-wide DNA methylation reprogramming in mouse primordial germ cells. *Molecular Cell*.

[21] Gkountela S., Li Z., Vincent J. J. (2013). The ontogeny of cKIT^+^ human primordial germ cells proves to be a resource for human germ line reprogramming, imprint erasure and *in vitro* differentiation. *Nature Cell Biology*.

[22] Ying Y., Qi X., Zhao G.-Q. (2002). Induction of primordial germ cells from pluripotent epiblast. *The Scientific World Journal*.

[23] Ohinata Y., Ohta H., Shigeta M., Yamanaka K., Wakayama T., Saitou M. (2009). A signaling principle for the specification of the germ cell lineage in mice. *Cell*.

[24] Ohinata Y., Payer B., O'Carroll D. (2005). Blimp1 is a critical determinant of the germ cell lineage in mice. *Nature*.

[25] West J. A., Viswanathan S. R., Yabuuchi A. (2009). A role for Lin28 in primordial germ-cell development and germ-cell malignancy. *Nature*.

[26] Mikedis M. M., Downs K. M. (2012). STELLA-positive subregions of the primitive streak contribute to posterior tissues of the mouse gastrula. *Developmental Biology*.

[27] Wei W., Qing T., Ye X. (2008). Primordial germ cell specification from embryonic stem cells. *PLoS ONE*.

[28] Hajkova P., Ancelin K., Waldmann T. (2008). Chromatin dynamics during epigenetic reprogramming in the mouse germ line. *Nature*.

[29] De Felici M. (2010). Germ stem cells in the mammalian adult ovary: considerations by a fan of the primordial germ cells. *Molecular Human Reproduction*.

[30] Ara T., Nakamura Y., Egawa T. (2003). Impaired colonization of the gonads by primordial germ cells in mice lacking a chemokine, stromal cell-derived factor-1 (SDF-1). *Proceedings of the National Academy of Sciences of the United States of America*.

[31] Zama A. M., Hudson F. P., Bedell M. A. (2005). Analysis of hypomorphic Kitl^SI^ mutants suggests different requirements for KITL in proliferation and migration of mouse primordial germ cells. *Biology of Reproduction*.

[32] Farini D., La Sala G., Tedesco M., De Felici M. (2007). Chemoattractant action and molecular signaling pathways of Kit ligand on mouse primordial germ cells. *Developmental Biology*.

[33] Takeuchi T., Tanigawa Y., Minamide R., Ikenishi K., Komiya T. (2010). Analysis of SDF-1/CXCR4 signaling in primordial germ cell migration and survival or differentiation in *Xenopus laevis*. *Mechanisms of Development*.

[34] Kimura T., Nakamura T., Murayama K. (2006). The stabilization of *β*-catenin leads to impaired primordial germ cell development via aberrant cell cycle progression. *Developmental Biology*.

[35] Saitou M., Kagiwada S., Kurimoto K. (2012). Epigenetic reprogramming in mouse pre-implantation development and primordial germ cells. *Development*.

[36] Surani M. A., Hajkova P. (2010). Epigenetic reprogramming of mouse germ cells toward totipotency. *Cold Spring Harbor Symposia on Quantitative Biology*.

[37] Kang J., Kalantry S., Rao A. (2013). PGC7, H3K9me2 and Tet3: regulators of DNA methylation in zygotes. *Cell Research*.

[38] Gu T.-P., Guo F., Yang H. (2011). The role of Tet3 DNA dioxygenase in epigenetic reprogramming by oocytes. *Nature*.

[39] Nakamura T., Arai Y., Umehara H. (2007). PGC7/Stella protects against DNA demethylation in early embryogenesis. *Nature Cell Biology*.

[40] Santos F., Peters A. H., Otte A. P., Reik W., Dean W. (2005). Dynamic chromatin modifications characterise the first cell cycle in mouse embryos. *Developmental Biology*.

[41] Bartolomei M. S., Ferguson-Smith A. C. (2011). Mammalian genomic imprinting. *Cold Spring Harbor Perspectives in Biology*.

[42] Baker T. G. (1963). A quantitative and cytological study of germ cells in human ovaries. *Proceedings of the Royal Society of London B: Biological Sciences*.

[43] Chen S.-R., Zheng Q.-S., Zhang Y., Gao F., Liu Y.-X. (2013). Disruption of genital ridge development causes aberrant primordial germ cell proliferation but does not affect their directional migration. *BMC Biology*.

[44] Reynolds N., Collier B., Maratou K. (2005). Dazl binds in vivo to specific transcripts and can regulate the pre-meiotic translation of Mvh in germ cells. *Human Molecular Genetics*.

[45] Gill M. E., Hu Y.-C., Lin Y., Page D. C. (2011). Licensing of gametogenesis, dependent on RNA binding protein DAZL, as a gateway to sexual differentiation of fetal germ cells. *Proceedings of the National Academy of Sciences of the United States of America*.

[46] Godin I., Deed R., Cooke J., Zsebo K., Dextert M., Wylie C. C. (1991). Effects of the steel gene product on mouse primordial germ cells in culture. *Nature*.

[47] Resnick J. L., Ortiz M., Keller J. R., Donovan P. J. (1998). Role of fibroblast growth factors and their receptors in mouse primordial germ cell growth. *Biology of Reproduction*.

[48] Kawase E., Hashimoto K., Pedersen R. A. (2004). Autocrine and paracrine mechanisms regulating primordial germ cell proliferation. *Molecular Reproduction and Development*.

[49] De Cuevas M., Lilly M. A., Spradling A. C. (1997). Germline cyst formation in *Drosophila*. *Annual Review of Genetics*.

[50] Pepling M. E. (2006). From primordial germ cell to primordial follicle: mammalian female germ cell development. *Genesis*.

[51] Kimble J. (2011). Molecular regulation of the mitosis/meiosis decision in multicellular organisms. *Cold Spring Harbor Perspectives in Biology*.

[52] Garcia M., Dietrich A. J. J., Freixa L., Vink A. C. G., Ponsa M., Egozcue J. (1987). Development of the first meiotic prophase stages in human fetal oocytes observed by light and electron microscopy. *Human Genetics*.

[53] Bowles J., Knight D., Smith C. (2006). Retinoid signaling determines germ cell fate in mice. *Science*.

[54] Choi Y.-J., Yoon J.-W., Pyo C.-W., Kim J.-A., Bae S.-H., Park S.-S. (2010). A possible role of STRA8 as a transcriptional factor. *Genes & Genomics*.

[55] Griswold M. D., Hogarth C. A., Bowles J., Koopman P. (2012). Initiating meiosis: the case for retinoic acid. *Biology of Reproduction*.

[56] Anderson E. L., Baltus A. E., Roepers-Gajadien H. L. (2008). Stra8 and its inducer, retinoic acid, regulate meiotic initiation in both spermatogenesis and oogenesis in mice. *Proceedings of the National Academy of Sciences of the United States of America*.

[57] Barrios F., Filipponi D., Pellegrini M. (2010). Opposing effects of retinoic acid and FGF9 on Nanos2 expression and meiotic entry of mouse germ cells. *Journal of Cell Science*.

[58] Lin Y., Gill M. E., Koubova J., Page D. C. (2008). Germ cell-intrinsic and -extrinsic factors govern meiotic initiation in mouse embryos. *Science*.

[59] Duester G. (2008). Retinoic acid synthesis and signaling during early organogenesis. *Cell*.

[60] Saba R., Kato Y., Saga Y. (2014). NANOS2 promotes male germ cell development independent of meiosis suppression. *Developmental Biology*.

[61] DiNapoli L., Capel B. (2008). SRY and the standoff in sex determination. *Molecular Endocrinology*.

[62] Tingen C., Kim A., Woodruff T. K. (2009). The primordial pool of follicles and nest breakdown in mammalian ovaries. *Molecular Human Reproduction*.

[63] Pepling M. E., Spradling A. C. (2001). Mouse ovarian germ cell cysts undergo programmed breakdown form primordial follicles. *Developmental Biology*.

[64] Wilkins A. S., Holliday R. (2009). The evolution of meiosis from mitosis. *Genetics*.

[65] Jones K. T. (2008). Meiosis in oocytes: predisposition to aneuploidy and its increased incidence with age. *Human Reproduction Update*.

[66] Adhikari D., Liu K. (2014). The regulation of maturation promoting factor during prophase I arrest and meiotic entry in mammalian oocytes. *Molecular and Cellular Endocrinology*.

[67] Godet M., Dametoy A., Mouradian S., Rudkin B. B., Durand P. (2004). Key role for cyclin-dependent kinases in the first and second meiotic divisions of rat spermatocytes. *Biology of Reproduction*.

[68] Malumbres M., Barbacid M. (2005). Mammalian cyclin-dependent kinases. *Trends in Biochemical Sciences*.

[69] Solc P., Schultz R. M., Motlik J. (2010). Prophase I arrest and progression to metaphase I in mouse oocytes: comparison of resumption of meiosis and recovery from G2-arrest in somatic cells. *Molecular Human Reproduction*.

[70] Solc P., Saskova A., Baran V., Kubelka M., Schultz R. M., Motlik J. (2008). CDC25A phosphatase controls meiosis I progression in mouse oocytes. *Developmental Biology*.

[71] Mehlmann L. M., Saeki Y., Tanaka S. (2004). The G_s_-linked receptor GPR3 maintains meiotic arrest in mammalian oocytes. *Science*.

[72] Sugiura K., Su Y.-Q., Diaz F. J. (2007). Oocyte-derived BMP15 and FGFs cooperate to promote glycolysis in cumulus cells. *Development*.

[73] De Felici M., Klinger F. G., Farini D., Scaldaferri M. L., Iona S., Lobascio M. (2005). Establishment of oocyte population in the fetal ovary: primordial germ cell proliferation and oocyte programmed cell death. *Reproductive BioMedicine Online*.

[74] Lobascio A. M., Klinger F. G., Scaldaferri M. L., Farini D., De Felici M. (2007). Analysis of programmed cell death in mouse fetal oocytes. *Reproduction*.

[75] Hutt K. J., McLaughlin E. A., Holland M. K. (2006). KIT/KIT ligand in mammalian oogenesis and folliculogenesis: Rroles in rabbit and murine ovarian follicle activation and oocyte growth. *Biology of Reproduction*.

[76] Pesce M., Farrace M. G., Piacentini M., Dolci S., De Felici M. (1993). Stem cell factor and leukemia inhibitory factor promote primordial germ cell survival by suppressing programmed cell death (apoptosis). *Development*.

[77] Jee B. C., Kim J. H., Park D. H., Youm H., Suh C. S., Kim S. H. (2012). In vitro growth of mouse preantral follicles: effect of animal age and stem cell factor/insulin-like growth factor supplementation. *Clinical and Experimental Reproductive Medicine*.

[78] Kim M.-R., Tilly J. L. (2004). Current concepts in Bcl-2 family member regulation of female germ cell development and survival. *Biochimica et Biophysica Acta*.

[79] De Felici M., Lobascio A. M., Klinger F. G. (2008). Cell death in fetal oocytes: many players for multiple pathways. *Autophagy*.

[80] Fulton N., Martins da Silva S. J., Bayne R. A. L., Anderson R. A. (2005). Germ cell proliferation and apoptosis in the developing human ovary. *Journal of Clinical Endocrinology and Metabolism*.

[81] Roudebush W. E., Kivens W. J., Mattke J. M. (2008). Biomarkers of ovarian reserve. *Biomarker Insights*.

[82] Mehlmann L. M., Kalinowski R. R., Ross L. F., Parlow A. F., Hewlett E. L., Jaffe L. A. (2006). Meiotic resumption in response to luteinizing hormone is independent of a G_i_ family G protein or calcium in the mouse oocyte. *Developmental Biology*.

[83] Perheentupa A., Huhtaniemi I. (2009). Aging of the human ovary and testis. *Molecular and Cellular Endocrinology*.

[84] Li Q., Geng X., Zheng W., Tamg J., Xu B., Shi Q. (2012). Current understanding of ovarian aging. *Science China Life Sciences*.

[85] Burt Solorzano C. M., McCartney C. R. (2010). Obesity and the pubertal transition in girls and boys. *Reproduction*.

[86] Bordini B., Rosenfield R. L. (2011). Normal pubertal development: part I: the endocrine basis of puberty. *Pediatrics in Review*.

[87] Parent A.-S., Teilmann G., Juul A., Skakkebaek N. E., Toppari J., Bourguignon J.-P. (2003). The timing of normal puberty and the age limits of sexual precocity: variations around the world, secular trends, and changes after migration. *Endocrine Reviews*.

[88] NHANES III (1997). *NHANES III Reference Manuals and Reports (CD-ROM). Analytic and Reporting Guidelines: The Third National Health and Nutrition Examination Survey (1988–94)*.

[89] Schoeters G., Den Hond E., Dhooge W., Van Larebeke N., Leijs M. (2008). Endocrine disruptors and abnormalities of pubertal development. *Basic and Clinical Pharmacology and Toxicology*.

[90] Macías-Tomei C., López-Blanco M., Espinoza I., Vasquez-Ramirez M. (2000). Pubertal development in caracas upper-middle-class boys and girls in a longitudinal context. *American Journal of Human Biology*.

[91] Bordini B., Rosenfield R. L. (2011). Normal pubertal development: part II: clinical aspects of puberty. *Pediatrics in Review*.

[92] Buck Louis G. M., Gray L. E., Marcus M. (2008). Environmental factors and puberty timing: expert panel research needs. *Pediatrics*.

[93] Özen S., Darcan Ş. (2011). Effects of environmental endocrine disruptors on pubertal development. *Journal of Clinical Research in Pediatric Endocrinology*.

[94] Rosenfield R. L., B.Lipton R., Drum M. L. (2009). Thelarche, pubarche, and menarche attainment in children with normal and elevated body mass index. *Pediatrics*.

[95] Huhtaniemi I. (1989). Endocrine function and regulation of the fetal and neonatal testis. *International Journal of Developmental Biology*.

[96] Huhtaniemi I. (1995). Molecular aspects of the ontogeny of the pituitary-gonodal axis. *Reproduction, Fertility and Development*.

[97] Grumbach M. M. (2002). The neuroendocrinology of human puberty revisited. *Hormone Research*.

[98] Terasawa E. (2005). Role of GABA in the mechanism of the onset of puberty in non-human primates. *International Review of Neurobiology*.

[99] Thind K. K., Goldsmith P. C. (1997). Expression of estrogen and progesterone receptors in glutamate and GABA neurons of the pubertal female monkey hypothalamus. *Neuroendocrinology*.

[100] Giuliani F. A., Escudero C., Casas S. (2013). Allopregnanolone and puberty: modulatory effect on glutamate and GABA release and expression of 3*α*-hydroxysteroid oxidoreductase in the hypothalamus of female rats. *Neuroscience*.

[101] Ojeda S. R., Dubay C., Lomniczi A. (2010). Gene networks and the neuroendocrine regulation of puberty. *Molecular and Cellular Endocrinology*.

[102] Ojeda S. R., Roth C., Mungenast A. (2006). Neuroendocrine mechanisms controlling female puberty: new approaches, new concepts. *International Journal of Andrology*.

[103] Funes S., Hedrick J. A., Vassileva G. (2003). The KiSS-1 receptor GPR54 is essential for the development of the murine reproductive system. *Biochemical and Biophysical Research Communications*.

[104] Seminara S. B., Messager S., Chatzidaki E. E. (2003). The *GPR54* gene as a regulator of puberty. *The New England Journal of Medicine*.

[105] d'Anglemont de Tassigny X., Colledge W. H. (2010). The role of Kisspeptin signaling in reproduction. *Physiology*.

[106] Rometo A. M., Krajewski S. J., Voytko M. L., Rance N. E. (2007). Hypertrophy and increased kisspeptin gene expression in the hypothalamic infundibular nucleus of postmenopausal women and ovariectomized monkeys. *Journal of Clinical Endocrinology and Metabolism*.

[107] Shahab M., Mastronardi C., Seminara S. B., Crowley W. F., Ojeda S. R., Plant T. M. (2005). Increased hypothalamic GPR54 signaling: a potential mechanism for initiation of puberty in primates. *Proceedings of the National Academy of Sciences of the United States of America*.

[108] Navarro V. M., Castellano J. M., Fernández-Fernández R. (2004). Developmental and hormonally regulated messenger ribonucleic acid expression of KiSS-1 and its putative receptor, GPR54, in rat hypothalamus and potent luteinizing hormone-releasing activity of KiSS-1 peptide. *Endocrinology*.

[109] Roa J., Aguilar E., Dieguez C., Pinilla L., Tena-Sempere M. (2008). New frontiers in kisspeptin/GPR54 physiology as fundamental gatekeepers of reproductive function. *Frontiers in Neuroendocrinology*.

[110] Smith J. T., Dungan H. M., Stoll E. A. (2005). Differential regulation of KiSS-1 mRNA expression by sex steroids in the brain of the male mouse. *Endocrinology*.

[111] Popa S. M., Clifton D. K., Steiner R. A. (2005). A KiSS to remember. *Trends in Endocrinology and Metabolism*.

[112] Plant T. M., Gay V. L., Marshall G. R., Arslan M. (1989). Puberty in monkeys is triggered by chemical stimulation of the hypothalamus. *Proceedings of the National Academy of Sciences of the United States of America*.

[113] Smith M. S. (2009). Estrus and menstrual cycles: neuroendocrine control. *Encyclopedia of Neuroscience*.

[114] Van Harmelen V., Reynisdottir S., Eriksson P. (1998). Leptin secretion from subcutaneous and visceral adipose tissue in women. *Diabetes*.

[115] Lammert A., Kiess W., Bottner A., Glasow A., Kratzsch J. (2001). Soluble leptin receptor represents the main leptin binding activity in human blood. *Biochemical and Biophysical Research Communications*.

[116] Smith J. T., Acohido B. V., Clifton D. K., Steiner R. A. (2006). KiSS-1 neurones are direct targets for leptin in the ob/ob mouse. *Journal of Neuroendocrinology*.

[117] Ahima R. S., Dushay J., Flier S. N., Prabakaran D., Flier J. S. (1997). Leptin accelerates the onset of puberty in normal female mice. *The Journal of Clinical Investigation*.

[118] Sepilian V. P., Crochet J. R., Nagamani M. (2006). Serum soluble leptin receptor levels and free leptin index in women with polycystic ovary syndrome: relationship to insulin resistance and androgens. *Fertility and Sterility*.

[119] Martos-Moreno G. Á., Chowen J. A., Argente J. (2010). Metabolic signals in human puberty: effects of over and undernutrition. *Molecular and Cellular Endocrinology*.

[120] Couzinet B., Young J., Brailly S., Le Bouc Y., Chanson P., Schaison G. (1999). Functional hypothalamic amenorrhoea: a partial and reversible gonadotrophin deficiency of nutritional origin. *Clinical Endocrinology*.

[121] Mantzoros C., Flier J. S., Lesem M. D., Brewerton T. D., Jimerson D. C. (1997). Cerebrospinal fluid leptin in anorexia nervosa: correlation with nutritional status and potential role in resistance to weight gain. *Journal of Clinical Endocrinology and Metabolism*.

[122] Argente J., Barrios I., Chowen J. A., Sinha M. K., Considine R. V. (1997). Leptin plasma levels in healthy Spanish children and adolescents, children with obesity, and adolescents anorexia nervosa and bulimia nervosa. *Journal of Pediatrics*.

[123] Muñoz M. T., Argente J. (2002). Anorexia nervosa in female adolescents: endocrine and bone mineral density disturbances. *European Journal of Endocrinology*.

[124] True C., Kirigiti M. A., Kievit P., Grove K. L., Smith M. S. (2011). Leptin is not the critical signal for kisspeptin or luteinising hormone restoration during exit from negative energy balance. *Journal of Neuroendocrinology*.

[125] Pralong F. P. (2010). Insulin and NPY pathways and the control of GnRH function and puberty onset. *Molecular and Cellular Endocrinology*.

[126] Gamba M., Pralong F. P. (2006). Control of GnRH neuronal activity by metabolic factors: the role of leptin and insulin. *Molecular and Cellular Endocrinology*.

[127] Hómez B. (2008). Hormonas en la mama: de la fisiología a la enfermedad. Revisión. *Revista Venezolana de Endocrinología y Metabolismo*.

[128] Anderson E. (2002). Progesterone receptors—animal models and cell signaling in breast cancer: the role of oestrogen and progesterone receptors in human mammary development and tumorigenesis. *Breast Cancer Research*.

[129] McBryan J., Howlin J., Napoletano S., Martin F. (2008). Amphiregulin: role in mammary gland development and breast cancer. *Journal of Mammary Gland Biology and Neoplasia*.

[130] Macias H., Hinck L. (2012). Mammary gland development. *Wiley Interdisciplinary Reviews: Developmental Biology*.

[131] Havelock J. C., Auchus R. J., Rainey W. E. (2004). The rise in adrenal androgen biosynthesis: adrenarche. *Seminars in Reproductive Medicine*.

[132] Kaludjerovic J., Ward W. E. (2012). The interplay between estrogen and fetal adrenal cortex. *Journal of Nutrition and Metabolism*.

[133] Nathan B. M., Palmert M. R. (2005). Regulation and disorders of pubertal timing. *Endocrinology and Metabolism Clinics of North America*.

[134] Völkl T. M. K., Öhl L., Rauh M., Schöfl C., Dörr H. G. (2011). Adrenarche and puberty in children with classic congenital adrenal hyperplasia due to 21-hydroxylase deficiency. *Hormone Research in Paediatrics*.

[135] Bocian-Sobkowska J., Woźniak W., Malendowicz L. K. (1998). Postnatal involution of the human adrenal fetal zone: stereologic description and apoptosis. *Endocrine Research*.

[136] Ishimoto H., Jaffe R. B. (2011). Development and function of the human fetal adrenal cortex: a key component in the feto-placental unit. *Endocrine Reviews*.

[137] Auchus R. J., Rainey W. E. (2004). Adrenarche-physiology, biochemistry and human disease. *Clinical Endocrinology*.

[138] Mellon S. H., Shively J. E., Miller W. L. (1991). Human proopiomelanocortin-(79–96), a proposed androgen stimulatory hormone, does not affect steroidogenesis in cultured human fetal adrenal cells. *Journal of Clinical Endocrinology and Metabolism*.

[139] Smith R., Mesiano S., Chan E.-C., Brown S., Jaffe R. B. (1998). Corticotropin-releasing hormone directly and preferentially stimulates dehydroepiandrosterone sulfate secretion by human fetal adrenal cortical cells. *Journal of Clinical Endocrinology and Metabolism*.

[140] Ong K. K., Potau N., Petry C. J. (2004). Opposing influences of prenatal and postnatal weight gain on adrenarche in normal boys and girls. *Journal of Clinical Endocrinology and Metabolism*.

[141] Fottner C., Engelhardt D., Weber M. M. (1998). Regulation of steroidogenesis by insulin-like growth factors (IGFs) in adult human adrenocortical cells: IGF-I and, more potently, IGF-II preferentially enhance androgen biosynthesis through interaction with the IGF-I receptor and IGF-binding proteins. *Journal of Endocrinology*.

[142] Biason-Lauber A., Zachmann M., Schoenle E. J. (2000). Effect of leptin on CYP17 enzymatic activities in human adrenal cells: new insight in the onset of adrenarche. *Endocrinology*.

[143] L’Allemand D., Schmidt S., Rousson V., Brabant G., Gasser T., Grüters A. (2002). Associations between body mass, leptin, IGF-I and circulating adrenal androgens in children with obesity and premature adrenarche. *European Journal of Endocrinology*.

[144] Xing Y., Parker C. R., Edwards M., Rainey W. E. (2010). ACTH is a potent regulator of gene expression in human adrenal cells. *Journal of Molecular Endocrinology*.

[145] Miller W. L., Auchus R. J. (2011). The molecular biology, biochemistry, and physiology of human steroidogenesis and its disorders. *Endocrine Reviews*.

[146] Akhtar M. K., Kelly S. L., Kaderbhai M. A. (2005). Cytochrome b_5_ modulation of 17*α* hydroxylase and 17–20 lyase (CYP17) activities in steroidogenesis. *Journal of Endocrinology*.

[147] Dharia S., Slane A., Jian M., Conner M., Conley A. J., Parker C. R. (2004). Colocalization of P450c17 and cytochrome b5 in androgen-synthesizing tissues of the human. *Biology of Reproduction*.

[148] Suzuki T., Sasano H., Takeyama J. (2000). Developmental changes in steroidogenic enzymes in human postnatal adrenal cortex: immunohistochemical studies. *Clinical Endocrinology*.

[149] Labrie F., Luu-The V., Labrie C., Pelletier G., El-Alfy M. (2000). Intracrinology and the skin. *Hormone Research*.

[150] Mihm M., Gangooly S., Muttukrishna S. (2011). The normal menstrual cycle in women. *Animal Reproduction Science*.

[151] Stricker R., Eberhart R., Chevailler M.-C., Quinn F. A., Bischof P., Stricker R. (2006). Establishment of detailed reference values for luteinizing hormone, follicle stimulating hormone, estradiol, and progesterone during different phases of the menstrual cycle on the Abbott ARCHITECT analyzer. *Clinical Chemistry and Laboratory Medicine*.

[152] Salha O., Abusheikha N., Sharma V. (2000). Dynamics of human follicular growth and in-vitro oocyte maturation. *Human Reproduction Update*.

[153] Baerwald A. R., Adams G. P., Pierson R. A. (2012). Ovarian antral folliculogenesis during the human menstrual cycle: a review. *Human Reproduction Update*.

[154] Sánchez F., Smitz J. (2012). Molecular control of oogenesis. *Biochimica et Biophysica Acta—Molecular Basis of Disease*.

[155] Findlay J. K., Drummond A. E. (1999). Regulation of the FSH receptor in the ovary. *Trends in Endocrinology and Metabolism*.

[156] Irving-Rodgers H. F., Morris S., Collett R. A. (2009). Phenotypes of the ovarian follicular basal lamina predict developmental competence of oocytes. *Human Reproduction*.

[157] Mihm M., Evans A. C. O. (2008). Mechanisms for dominant follicle selection in monovulatory species: a comparison of morphological, endocrine and intraovarian events in cows, mares and women. *Reproduction in Domestic Animals*.

[158] Miller W., Geller D., Rosen M. (2007). Ovarian and adrenal androgen biosynthesis and metabolism. *Androgen Excess Disorders in Women*.

[159] Jonard S., Dewailly D. (2004). The follicular excess in polycystic ovaries, due to intra-ovarian hyperandrogenism, may be the main culprit for the follicular arrest. *Human Reproduction Update*.

[160] Erickson G. F., Shimasaki S. (2001). The physiology of folliculogenesis: the role of novel growth factors. *Fertility and Sterility*.

[161] Rodgers R. J., Irving-Rodgers H. F. (2010). Formation of the ovarian follicular antrum and follicular fluid. *Biology of Reproduction*.

[162] Erickson G. F., Magoffin D. A., Dyer C. A., Hofeditz C. (1985). The ovarian androgen producing cells: a review of structure/function relationships. *Endocrine Reviews*.

[163] Zeleznik A. J. (2004). The physiology of follicle selection. *Reproductive Biology and Endocrinology*.

[164] Roseff S. J., Bangah M. L., Kettel L. M. (1989). Dynamic changes in circulating inhibin levels during the luteal-follicular transition of the human menstrual cycle. *The Journal of Clinical Endocrinology & Metabolism*.

[165] Son W.-Y., Das M., Shalom-Paz E., Holzer H. (2011). Mechanisms of follicle selection and development. *Minerva Ginecologica*.

[166] Miyazono K., Kamiya Y., Morikawa M. (2010). Bone morphogenetic protein receptors and signal transduction. *Journal of Biochemistry*.

[167] Thompson T. B., Cook R. W., Chapman S. C., Jardetzky T. S., Woodruff T. K. (2004). Beta A versus beta B: is it merely a matter of expression?. *Molecular and Cellular Endocrinology*.

[168] Aleman-Muench G. R., Soldevila G. (2012). When versatility matters: activins/inhibins as key regulators of immunity. *Immunology and Cell Biology*.

[169] McNeilly A. S. (2012). Diagnostic applications for inhibin and activins. *Molecular and Cellular Endocrinology*.

[170] Knight P. G., Satchell L., Glister C. (2012). Intra-ovarian roles of activins and inhibins. *Molecular and Cellular Endocrinology*.

[171] El-Roeiy A., Chen X., Roberts V. J., LeRoith D., Roberts C. T., Yen S. S. C. (1993). Expression of insulin-like growth factor-I (IGF-I) and IGF-II and the IGF-I, IGF-II, and insulin receptor genes and localization of the gene products in the human ovary. *Journal of Clinical Endocrinology and Metabolism*.

[172] Findlay J. K. (1993). An update on the roles of inhibin, activin, and follistatin as local regulators of folliculogenesis. *Biology of Reproduction*.

[173] Knight P. G., Glister C. (2006). TGF-*β* superfamily members and ovarian follicle development. *Reproduction*.

[174] Thompson T. B., Lerch T. F., Cook R. W., Woodruff T. K., Jardetzky T. S. (2005). The structure of the follistatin: activin complex reveals antagonism of both type I and type II receptor binding. *Developmental Cell*.

[175] Glister C., Groome N. P., Knight P. G. (2006). Bovine follicle development is associated with divergent changes in activin-A, inhibin-A and follistatin and the relative abundance of different follistatin isoforms in follicular fluid. *Journal of Endocrinology*.

[176] Glister C., Satchell L., Knight P. G. (2010). Changes in expression of bone morphogenetic proteins (BMPs), their receptors and inhibin co-receptor betaglycan during bovine antral follicle development: inhibin can antagonize the suppressive effect of BMPs on thecal androgen production. *Reproduction*.

[177] Young J. M., McNeilly A. S. (2010). Theca: the forgotten cell of the ovarian follicle. *Reproduction*.

[178] Groothuis P. G., Dassen H. H. N. M., Romano A., Punyadeera C. (2007). Estrogen and the endometrium: lessons learned from gene expression profiling in rodents and human. *Human Reproduction Update*.

[179] Bromer J. G., Aldad T. S., Taylor H. S. (2009). Defining the proliferative phase endometrial defect. *Fertility and Sterility*.

[180] Tomikawa J., Uenoyama Y., Ozawa M. (2012). Epigenetic regulation of *Kiss1* gene expression mediating estrogen-positive feedback action in the mouse brain. *Proceedings of the National Academy of Sciences of the United States of America*.

[181] Jackson J. A., Tischkau S. A., Zhang P., Bahr J. M. (1994). Plasminogen activator production by the granulosa layer is stimulated by factor(s) produced by the theca layer and inhibited by the luteinizing hormone surge in the chicken. *Biology of Reproduction*.

[182] Reich R., Miskin R., Tsafriri A. (1985). Follicular plasminogen activator: involvement in ovulation. *Endocrinology*.

[183] Murdoch W. J., McDonnel A. C. (2002). Roles of the ovarian surface epithelium in ovulation and carcinogenesis. *Reproduction*.

[184] Brannstrom M., Bonello N., Wang L. J., Norman R. J. (1995). Effects of tumour necrosis factor alpha (TNF alpha) on ovulation in the rat ovary. *Reproduction, Fertility and Development*.

[185] Gómez R., Simón C., Remohí J., Pellicer A. (2003). Administration of moderate and high doses of gonadotropins to female rats increases ovarian vascular endothelial growth factor (VEGF) and VEGF receptor-2 expression that is associated to vascular hyperpermeability. *Biology of Reproduction*.

[186] Russell D. L., Robker R. L. (2007). Molecular mechanisms of ovulation: co-ordination through the cumulus complex. *Human Reproduction Update*.

[187] Peng X.-R., Hsueh A. J. W., LaPolt P. S., Bjersing L., Ny T. (1991). Localization of luteinizing hormone receptor messenger ribonucleic acid expression in ovarian cell types during follicle development and ovulation. *Endocrinology*.

[188] Norris R. P., Ratzan W. J., Freudzon M. (2009). Cyclic GMP from the surrounding somatic cells regulates cyclic AMP and meiosis in the mouse oocyte. *Development*.

[189] Kalinowski R. R., Jaffe L. A., Foltz K. R., Giusti A. F. (2003). A receptor linked to a Gi-family G-protein functions in initiating oocyte maturation in starfish but not frogs. *Developmental Biology*.

[190] Mehlmann L. M., Jones T. L. Z., Jaffe L. A. (2002). Meiotic arrest in the mouse follicle maintained by a Gs protein in the oocyte. *Science*.

[191] Ma C., Benink H. A., Cheng D. (2006). Cdc42 activation couples spindle positioning to first polar body formation in oocyte maturation. *Current Biology*.

[192] Zhang X., Ma C., Miller A. L., Katbi H. A., Bement W. M., Liu X. J. (2008). Polar body emission requires a RhoA contractile ring and Cdc42-mediated membrane protrusion. *Developmental Cell*.

[193] Oh J. S., Susor A., Schindler K., Schultz R. M., Conti M. (2013). Cdc25A activity is required for the metaphase II arrest in mouse oocytes. *Journal of Cell Science*.

[194] Oh J. S., Susor A., Conti M. (2011). Protein tyrosine kinase Wee1B is essential for metaphase II exit in mouse oocytes. *Science*.

[195] Chang H.-Y., Jennings P. C., Stewart J., Verrills N. M., Jones K. T. (2011). Essential role of protein phosphatase 2A in metaphase II arrest and activation of mouse eggs shown by okadaic acid, dominant negative protein phosphatase 2A, and FTY720. *The Journal of Biological Chemistry*.

[196] Christian C. A., Moenter S. M. (2010). The neurobiology of preovulatory and estradiol-induced gonadotropin-releasing hormone surges. *Endocrine Reviews*.

[197] Gaytán M., Bellido C., Morales C., Sánchez-Criado J. E., Gaytán F. (2006). Effects of selective inhibition of cyclooxygenase and lipooxygenase pathways in follicle rupture and ovulation in the rat. *Reproduction*.

[198] Markosyan N., Duffy D. M. (2009). Prostaglandin E2 acts via multiple receptors to regulate plasminogen-dependent proteolysis in the primate periovulatory follicle. *Endocrinology*.

[199] Bachelot A., Binart N. (2005). Corpus luteum development: lessons from genetic models in mice. *Current Topics in Developmental Biology*.

[200] Smith M. F., McIntush E. W., Smith G. W. (1994). Mechanisms associated with corpus luteum development. *Journal of Animal Science*.

[201] Niswender G. D. (2002). Molecular control of luteal secretion of progesterone. *Reproduction*.

[202] Richards R. G., Gadsby J. E., Almond G. W. (1994). Differential effects of LH and PGE2 on progesterone secretion by small and large porcine luteal cells. *Journal of Reproduction and Fertility*.

[203] Smith C. J., Sridaran R. (1989). The steroidogenic response of large and small luteal cells to dibutyryl Camp and 25-OH-cholesterol. *Growth Factors and the Ovary*.

[204] Ottander U., Poromaa I. S., Bjurulf E., Skytt Å., Bäckström T., Olofsson J. I. (2005). Allopregnanolone and pregnanolone are produced by the human corpus luteum. *Molecular and Cellular Endocrinology*.

[205] Genazzani A. R., Palumbo M. A., De Micheroux A. A. (1995). Evidence for a role for the neurosteroid allopregnanolone in the modulation of reproductive function in female rats. *European Journal of Endocrinology*.

[206] Shirasuna K., Shimizu T., Matsui M., Miyamoto A. (2013). Emerging roles of immune cells in luteal angiogenesis. *Reproduction, Fertility and Development*.

[207] García-Pascual C. M., Zimmermann R. C., Ferrero H. (2013). Delta-like ligand 4 regulates vascular endothelial growth factor receptor 2-driven luteal angiogenesis through induction of a tip/stalk phenotype in proliferating endothelial cells. *Fertility and Sterility*.

[208] Mokrý J., Čížková D., Filip S. (2004). Nestin expression by newly formed human blood vessels. *Stem Cells and Development*.

[209] Weems Y. S., Lennon E., Uchima T. (2005). Is nitric oxide luteolytic or antiluteolytic?. *Prostaglandins & Other Lipid Mediators*.

[210] Hawkins S. M., Matzuk M. M. (2008). Menstrual cycle: basic biology. *Annals of the New York Academy of Sciences*.

[211] Lindaman A., Dowden A., Zavazava N. (2006). Soluble HLA-G molecules induce apoptosis in natural killer cells. *American Journal of Reproductive Immunology*.

[212] Gutiérrez A., Donato R., Mindlin A. (2006). Aspectos inmunológicos del embarazo normal. *Archivos de Alergia e Inmunología Clínica*.

[213] Roughton S. A., Lareu R. R., Bittles A. H., Dharmarajan A. M. (1999). Fas and Fas ligand messenger ribonucleic acid and protein expression in the rat corpus luteum during apoptosis-mediated luteolysis. *Biology of Reproduction*.

[214] Das M., Djahanbakhch O., Hacihanefioglu B. (2008). Granulosa cell survival and proliferation are altered in polycystic ovary syndrome. *Journal of Clinical Endocrinology and Metabolism*.

[215] Amsterdam A., Sasson R., Keren-Tal I. (2003). Alternative pathways of ovarian apoptosis: death for life. *Biochemical Pharmacology*.

[216] Focchi G. R., Simões M. J., Baracat E. C., de Lima G. R. (1995). Morphological and morphometrical features of the corpus albicans in the course of the postmenopausal period. *Bulletin de l"Association des Anatomistes*.

[217] Joel R. V., Foraker A. G. (1960). Fate of the corpus albicans: a morphologic approach. *American Journal of Obstetrics & Gynecology*.

[218] Salamonsen L. A., Dimitriadis E., Jones R. L., Nie G. (2003). Complex regulation of decidualization: a role for cytokines and proteases. A review. *Placenta*.

[219] Schindler A. E. (2005). Endocrinology of pregnancy: consequences for the diagnosis and treatment of pregnancy disorders. *Journal of Steroid Biochemistry and Molecular Biology*.

[220] Burger H. G. (1996). The endocrinology of the menopause. *Maturitas*.

[221] Santoro N., Isaac B., Neal-Perry G. (2003). Impaired folliculogenesis and ovulation in older reproductive aged women. *Journal of Clinical Endocrinology and Metabolism*.

[222] Burger H. G., Hale G. E., Dennerstein L., Robertson D. M. (2008). Cycle and hormone changes during perimenopause: the key role of ovarian function. *Menopause*.

[223] Hall J. E., Lavoie H. B., Marsh E. E., Martin K. A. (2000). Decrease in gonadotropin-releasing hormone (GnRH) pulse frequency with aging in postmenopausal women. *Journal of Clinical Endocrinology and Metabolism*.

[224] Downs J. L., Wise P. M. (2009). The role of the brain in female reproductive aging. *Molecular and Cellular Endocrinology*.

[225] Morita Y., Tilly J. L. (1999). Oocyte apoptosis: like sand through an hourglass. *Developmental Biology*.

[226] Stocco C. (2012). Tissue physiology and pathology of aromatase. *Steroids*.

[227] Warburton D. E. R., Nicol C. W., Gatto S. N., Bredin S. S. D. (2007). Cardiovascular disease and osteoporosis: balancing risk management. *Vascular Health and Risk Management*.

[228] Ellis K. J., Nicolson M. (1997). Leptin levels and body fatness in children: effects of gender, ethnicity, and sexual development. *Pediatric Research*.

[229] Bandini L. G., Must A., Naumova E. N. (2008). Change in leptin, body composition and other hormones around menarche—a visual representation. *Acta Paediatrica*.

[230] Yüksel B., Özbek M. N., Mungan N. Ö. (2011). Serum IGF-1 and IGFBP-3 levels in healthy children between 0 and 6 years of age. *Journal of Clinical Research in Pediatric Endocrinology*.

